# Genomic Analysis Reveals a New Cryptic Taxon Within the *Anopheles gambiae* Complex With a Distinct Insecticide Resistance Profile in the Coast of East Africa

**DOI:** 10.1111/mec.17762

**Published:** 2025-04-16

**Authors:** Sophia H. Mwinyi, Kelly L. Bennett, Sanjay C. Nagi, Bilali Kabula, Johnson Matowo, David Weetman, Francesco Baldini, Simon A. Babayan, Martin J. Donnelly, Chris S. Clarkson, Fredros O. Okumu, Alistair Miles

**Affiliations:** ^1^ Environmental Health and Ecological Sciences Department Ifakara Health Institute Ifakara Morogoro Tanzania; ^2^ School of Biodiversity, One Health and Veterinary Medicine University of Glasgow Glasgow UK; ^3^ Genomic Surveillance Unit Wellcome Sanger Institute Cambridge UK; ^4^ Department of Vector Biology Liverpool School of Tropical Medicine Liverpool UK; ^5^ National Institute for Medical Research (NIMR), Amani Centre Muheza Tanzania; ^6^ Kilimanjaro Christian Medical University College (KCMUCo), Tumaini University Moshi Tanzania; ^7^ School of Life Science and Biotechnology Nelson Mandela African Institution of Science and Technology Arusha Tanzania

**Keywords:** *Anopheles gambiae* complex, cryptic species, insecticide resistance, population structure, whole‐genome sequencing

## Abstract

*Anopheles* mosquitoes are major malaria vectors, encompassing several species complexes with diverse life histories, transmission risks and insecticide resistance profiles that challenge malaria control efforts. This study investigated the genetic structure and insecticide resistance profiles of *Anopheles gambiae* complex mosquitoes in Tanzania. We analysed whole‐genome sequence data of 300 mosquitoes collected between 2012 and 2015 across four regions in northern Tanzania and identified *An. gambiae* s.s., *An. arabiensis* and a distinct taxonomic group that was previously unknown. This distinct taxon has a unique profile of genetic diversity and appears restricted to the coastal region, and we refer to it as the *Pwani* molecular form. Analysis of insecticide resistance based on target‐site mutations and copy number variations (CNV) showed that these markers were strikingly absent from the *Pwani* molecular form in contrast to other taxa. Our analysis also revealed a pattern of geographical isolation in the *An. gambiae* s.s. populations, with samples from the north‐western site (Muleba) clustering separately from those collected in the north‐eastern site (Muheza). These geographically isolated subpopulations also had differing resistance and selection profiles, with *An. gambiae* s.s. from the north‐western site showing genomic evidence of higher resistance to pyrethroids compared with the north‐eastern population. Conversely, *An. arabiensis* showed no geographical population structuring, with a similar insecticide resistance profile across all sampling locations, suggesting unrestricted gene flow. Our findings underscore the need to incorporate genetic data into malaria vector surveillance and control decisions and could inform the development and deployment of new interventions.

**Abbreviations**
AIMs: ancestry informative marker
BWA: Burrows–Wheeler alignment
CDS: codon DNA sequence
CNV: copy number variation
DDT: dichloro‐diphenyl‐trichloroethane
DNA: deoxyribonucleic acid
GABA: gamma‐aminobutyric acid
GSTe: glutathione S‐transferase genes
GWSS: genome‐wide selection scans
IRS: indoor residual spray
ITNs: insecticide‐treated nets
Kdr: knock‐down resistance
LLINs: long lasting insecticide nets
NJT: neighbour joining trees
PCA: principal component analysis
SNP: single nucleotide polymorphism
VGSC: voltage‐gated sodium channel
WGS: whole genome sequencing

## 1 Introduction

The insect order *Diptera* has the largest number of disease vectors (Vieira et al. 2024), which together contribute to the transmission of over 17% of all infectious diseases and more than 700,000 deaths annually (World Health Organization 2020). In recent decades, numerous insect populations have been identified to be cryptic species (Busvine 1980). Despite morphological similarities, cryptic vector species can exhibit different physiological, biological and ecological behaviours that impact disease transmission and their response to control interventions (Busvine 1980). Additional genetic complexity may result from geographical barriers and varying environmental conditions, influenced by human activities or climate, which create variability within species and, over time, can lead to further speciation (Busvine 1980; Ostfeld et al. 2010). Understanding cryptic species distributions across the landscape, including how populations are connected by gene flow and share genomic variants that allow them to escape vector control, is of increasing importance for disease control programmes.

The challenge of cryptic species is of marked importance in the *Anopheles gambiae* complex. Once considered a single species (Fontaine et al. 2015), the *An. gambiae* complex is currently believed to be composed of at least 10 morphologically indistinguishable sibling species, with three newly discovered cryptic taxa in West Africa over the past decade (Tennessen et al. 2021; Crawford et al. 2016; Barrón et al. 2019). In Tanzania, the two primary vectors within the group, *An. gambiae* s.s. (hereafter referred to as *An. gambiae*) and *An. arabiensis* (White 1974), differ significantly in their ecology. *An. gambiae* exhibits high levels of anthropophagic and endophagic behaviour (White 1974; Coluzzi et al. 1979), whereas *An. arabiensis* tends to feed more readily on animals outdoors (Coluzzi et al. 2002; Gillies and De Meillon 1968; White et al. 1972). Additionally, *An. gambiae* is more prevalent in humid coastal and lacustrine regions, while *An. arabiensis* is more common in dry and semi‐arid areas (Mnzava and Kilama 1986). Studies have also reported the presence of two minor vectors in East Africa: *An. merus*, a salt‐water breeding member known to transmit malaria and lymphatic filariasis (Temu et al. 1998; Mosha 1983; Bushrod 1981) and *An. quadriannulatus*, both of which exhibit exophagic, exophilic and zoophagic behaviours (White 1974). Previous studies suggested the absence or low likelihood of finding *An. coluzzii* in Eastern Africa (Sinka et al. 2020; Della Torre et al. 2005; Santolamazza et al. 2015), although this is now negated by the recent discovery of resident populations of *An. coluzzii* in Northern Kenya (Kamau et al. 2024).

Environmental, ecological and physiological adaptations, influenced by climate and human activities such as land use, significantly influence the spatial distribution and genetic diversity of *Anopheles* vector populations (Mnzava and Kilama 1986; Koffi et al. 2023; Castro 2017; Winskill et al. 2011; Rumisha et al. 2019; Mboera et al. 2015; Ijumba et al. 2002; Stryker and Bomblies 2012; Chan et al. 2022). In addition, isolating factors such as geographic distance and physical barriers may play a crucial role in restricting dispersal and gene flow, potentially leading to pronounced population substructures within species (Busvine 1980; Touré et al. 1994, 1998; Della Torre et al. 2002; Donnelly and Townson 2000; Simard et al. 1999; Nyanjom et al. 2003; Besansky et al. 1997). For instance, *An. gambiae* populations exhibit pronounced genetic structure across major geographical barriers such as the East African Rift Valley, but these have minimal impact on gene flow in *An. arabiensis* (Ng'habi et al. 2011). Limited studies focusing on the genetic structure of *An*. *gambiae* and *An*. *arabiensis* have been conducted in Tanzania (Ng'habi et al. 2011; Gélin et al. 2016; O'Loughlin et al. 2016; Matowo et al. 2014). For example, a study conducted in Dar es Salaam and Bagamoyo using microsatellite markers found high genetic differentiation between *An. gambiae* from nearby locations but weak differentiation between *An. arabiensis* driven by isolation‐by‐distance (Maliti et al. 2014). Conversely, a similar study conducted in southeastern Tanzania, in the Kilombero valley revealed high levels of genetic differentiation in *An. arabiensis* but not *An. gambiae* populations, possibly due to ecological diversification rather than physical barriers or distance (Ng'habi et al. 2011). These differences in population structure can influence the insecticide resistance profiles of mosquito populations, impacting the effectiveness of malaria control measures.

In sub‐Saharan Africa, malaria control heavily relies on vector control interventions like insecticide‐treated nets (ITNs) and indoor residual spraying (IRS) (Pluess et al. 2010). However, their effectiveness is severely challenged by the evolution of insecticide resistance. Several studies in Tanzania reported resistance in the *An. gambiae* complex to four chemical classes: pyrethroids, organophosphates, carbamates and organochlorines (Kabula et al. 2012, 2014; Nkya et al. 2014; Matowo et al. 2010; Kisinza et al. 2017). Insecticide resistance in malaria vectors primarily stems from mutations in insecticide target sites and enhanced metabolism due to elevated expression or upregulation of detoxifying enzymes (Schmidt et al. 2010; Hemingway 2014). Notable target sites include the voltage‐gated sodium channel (*Vgsc*) gene affected by pyrethroids, the acetylcholinesterase (*Ace‐1*) gene impacted by carbamates and organophosphates, and the GABA receptor subunit encoded by the *Rdl* (resistant to dieldrin) gene (Elanga‐Ndille et al. 2019; Du et al. 2005; Clarkson et al. 2021). Additionally, key metabolic resistance genes include cytochrome P450 monooxygenases (*P450s*), carboxylesterases (*Coe*), and glutathione S‐transferases (*Gst*) (Lucas et al. 2019; N'Dri et al. 2023; Liu 2015). Studies also suggest that species such as *An. arabiensis* that readily feed outdoors may have lower resistance than indoor‐biting species like *An. gambiae* (Mawejje et al. 2023) and *An. funestus* (Pinda et al. 2020).

Whole‐genome studies offer a higher resolution compared to single or limited marker studies for identifying species (Wang et al. 2019), determining population structure (Tennessen et al. 2021; Logue et al. 2015), detecting insecticide resistance variants (Lucas et al. 2019; Kientega et al. 2023; Nagi et al. 2024) and enhancing our understanding of complex evolutionary traits (Akoniyon et al. 2022). A genomic perspective may enable improved vector control strategies that are species‐specific and sensitive to the presence of insecticide resistance mutations. Therefore, this study analysed whole‐genome sequence data of *An. gambiae* complex mosquitoes collected from four sites spanning northwestern to northeastern Tanzania to address two key questions. First, we assess the taxonomic status of *An. gambiae* complex collected from Tanzania. Secondly, we test the hypothesis that there is geographical structure and variation in insecticide resistance profiles of both *An. gambiae* and *An. arabiensis* in Tanzania.

## 2 Materials and Methods

### 2.1 Mosquito Collection

**FIGURE 1:**
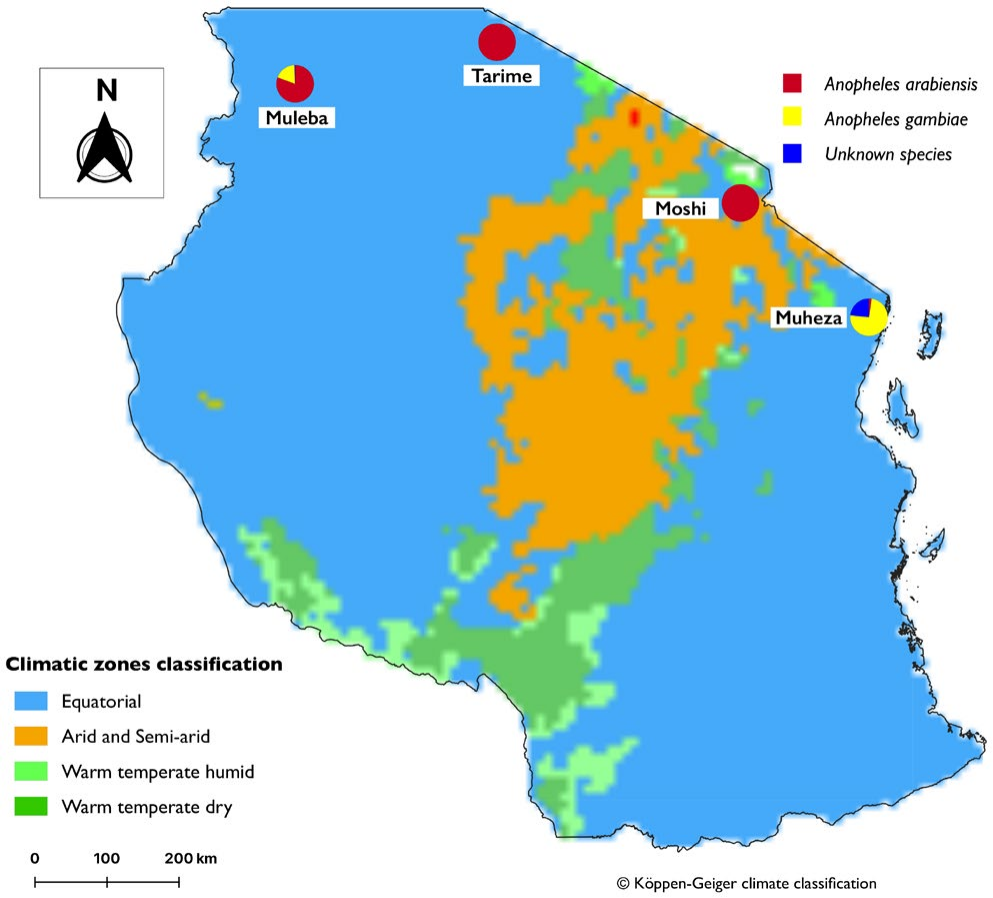
Map of Tanzania showing mosquito collection sites with the distribution of provisional species assigned using ancestry informative markers (AIMs) in each region and the Köppen climate classification in Tanzania.

This study used data from the *An. gambiae* 1000 Genomes Project (Ag1000G) Phase 3 (3.0 release), comprising mosquitoes from Tanzania that were collected in four regions, Muheza and Moshi in north east, and Muleba and Tarime in the north‐western regions (Figure 1).

In Muheza, samples were collected inside randomly selected homes during the evening hours from Zeneti village (−5.217° S, 38.650° E) between November 2012 and May 2013 using indoor‐resting collections and pyrethrum spray catches (Kabula et al. 2016). Muheza district is known to be highly prone to seasonal malaria transmission, peaking after the rainy season in May and June (Kabula et al. 2012; Makenga et al. 2022). Samples from Moshi were collected as larvae between August and September 2012 from rice fields in the lower Mabogini area (−3.400° S, 37.350° E) on the southern slopes of Mount Kilimanjaro (Matowo et al. 2014). After rearing larvae to adults, females were exposed to a variety of pesticides (permethrin, lambda cyhalothrin, fenitrothion, dichloro‐diphenyl‐trichloroethane (DDT) and bendiocarb) for susceptibility tests prior to sequencing. These tests revealed full susceptibility to all the insecticides as previously reported (Matowo et al. 2014). In addition, larvae were collected from Tarime in Komaswa village (−1.417° S, 34.183° E) in August 2012 and raised to adults for sequencing. Similarly, sampling was conducted in Muleba district (1.750° S, 31.667° E) in 2015 in six villages over both dry and wet seasons in a 6‐month period. Large‐scale malaria control efforts began in 2006 in this district, with the introduction of Indoor Residual Spraying (IRS) operations, with the use of pyrethroid (lambda cyhalothrin), followed by carbamate (bendiocarb) in 2010, and ultimately the organophosphate, pirimiphos methyl (Actellic 300SC) in 2014 (Tungu et al. 2023). These operations coincided with the expansion of long‐lasting insecticidal nets (LLINs) through universal coverage in the lake zone region.

### 2.2 Data Processing

#### 2.2.1 Sequencing and Single Nucleotide Polymorphisms (SNP) Calling

Sequencing and SNP calling was performed according to the Ag1000G Phase 3 project protocols developed from Phase 1 and 2 protocols (Clarkson et al. 2020; Miles et al. 2017). The alignment and SNP calling protocols are available online (see protocol). Briefly, paired‐end multiplex libraries were processed using Illumina's deoxyribonucleic acid (DNA) preparation protocol, with the exception that genomic DNA was fragmented using Covaris Adaptive Focused Acoustics rather than nebulisation. Multiplexes comprised 12 tagged individual mosquitoes. Three lanes of sequencing were generated for each multiplex to even out variations in yield between sequencing runs. The samples were sequenced to an average coverage of 30× using either the Illumina HiSeq 2000 or Illumina HiSeq X platform, yielding an insert size between 100 and 200 bp.

Reads were aligned to the AgamP4 reference genome using the Burrows–Wheeler alignment algorithm (BWA) version 0.7.15 (Li and Durbin 2009). Indel realignment was performed using GATK version 3.7.0 RealignerTargetCreator and IndelRealigner (McKenna et al. 2010). Single nucleotide polymorphisms were called using GATK version 3.7.0 UnifiedGenotyper (McKenna et al. 2010). Genotypes were called for each sample independently against a set of given alleles based on the reference genome, in genotyping mode, given all possible alleles at all genomic sites where the reference base was not defined (‘N’). To manage the data quantity, samples with excess coverage were down sampled to 250× within the UnifiedGenotyper.

#### 2.2.2 Quality Control of Whole‐Genome Sequencing

Quality control of whole‐genome sequencing data was carried out as described by the Ag1000G phase 3 project developed during previous phases. Samples were excluded if median coverage across all chromosomes was less than 10×, or if less than 80% of the reference genome was covered by at least 1×. Median coverage was used to minimise the impact of repetitive genomic regions with excessively high or low coverage, because it is less sensitive to outliers. A model for detecting contamination in NGS alignments previously described by Jun et al. (2012) was used to identify and exclude samples affected by cross‐contamination. Briefly, the method estimates the likelihood of the observed alternate and reference allele counts under different contamination fractions, given approximate population allele frequencies. Population allele frequencies were estimated from the Ag1000G Phase 2 data release (Clarkson et al. 2020). The model computes maximum likelihood values for *alpha*, representing percentage contamination. Samples were excluded if *alpha* was 4.5% or greater. Some samples were sequenced a second time if the first sequencing run did not obtain adequate yield. Following Ag1000G Phase 3 protocols, average pairwise genetic distance was computed between sample pairs using city block distance between the genotype allele counts, excluding sites with missing data, using custom scripts. All replicates for a given sample were excluded if the genetic distance exceeded 0.006; else one member was retained.

Site filters defined by the Ag1000G project were applied to exclude sites where SNP calling and genotyping were less reliable. Site filters were generated using the 15 colony crosses in the Ag1000G Phase 3 data set, comprising two parents and up to 20 progenies. These data were used to identify genotypes that were not consistent with Mendelian inheritance and so likely to result from sequencing, alignment or SNP calling errors. Five crosses were retained for validation, and the remaining 10 were used to identify a positive training set comprised of sites with no Mendelian errors across all crosses, or a negative training set where at least one error was observed. A balanced training set was also generated containing 100,000 autosomal sites from each of the positive and negative training sets. A decision tree model was then trained using the generated data sets along with input summary statistics based on the coverage, genotype quality and mapping quality of the wild‐caught samples. Sites across the genome were assigned as either passing or failing site filters based on the resulting decision tree model.

#### 2.2.3 Haplotype Phasing

Genotypes at biallelic SNPs passing site filters were phased into haplotypes using a combination of read‐backed and statistical phasing as defined by the Ag1000G Phase 3 project available online (haplotype‐phasing). Read‐backed phasing was performed for each individual using WhatsHap version 1.0 (Martin et al. 2022) and statistical phasing was done using SHAPEIT v 4.2.1 (Delaneau and Marchini 2014).

#### 2.2.4 Species Calling Using Ancestry‐Informative Markers

Ancestry‐informative markers (AIMs) from the *Anopheles* 16 genomes project (Neafsey et al. 2015) were used to distinguish *An. arabiensis* from other taxa. Alleles were mapped onto the same alternate allele space, and allele frequencies were computed for both species. Sites that were multiallelic in either group or had missing data were excluded. A total of 565,329 SNPs were identified as potentially informative, where no shared alleles were present between groups. *An. gambiae* and *An. coluzzii* were distinguished from one another using AIMs generated by the Ag1000G Phase 2 project. A set of 2612 AIMs was used to differentiate *An. gambiae*/*An. coluzzii* from *An. arabiensis*, and 700 AIMs were used to differentiate *An. gambiae* from *An. coluzzii*. Individuals were classified as *An. arabiensis* when the fraction of arabiensis‐like alleles was greater than 0.6; otherwise, they were classified as *An. gambiae*. Individuals were called as *An. gambiae* where the fraction of coluzzii‐like calls was < 0.12 and as *An. coluzzii* where this fraction was > 0.90. Individuals in‐between these fractions were classified as intermediate.

#### 2.2.5 Copy Number Variants (CNV) Calling

CNV calling was conducted following the methodology outlined by Lucas et al. (2019). The copy number state across genomic windows for each individual sample was derived from normalised coverage data using a Gaussian Hidden Markov model (HMM), implemented by hmmlearn (https://github.com/hmmlearn/hmmlearn). A CNV call was defined as a contiguous sequence of at least five genomic windows with a predicted copy number > 2, or > 1 for the X chromosome in males. CNV calls were filtered to retain those with a likelihood score greater than 1000 as estimated by the HMM. Additionally, individuals exhibiting a coverage variance greater than 0.2 were excluded from the analysis to enhance the reliability of the CNV calls.

### 2.3 Data Analysis

#### 2.3.1 Population Structure and Genetic Diversity

The population structure of the Tanzanian mosquito samples was investigated by first conducting a principal component analysis (PCA) and unrooted neighbour‐joining tree (NJT) across the 3L chromosome arm, which was chosen because it is unaffected by large structural rearrangements such as inversions. The analysis used 100,000 biallelic SNPs equally distributed across the chromosome arm with a minor allele count greater than 2 and with no missing calls. To investigate whether the taxonomic structure we observed in Tanzania extends across East Africa, a PCA and NJT were also performed including samples from neighbouring Kenya and Uganda available in the Ag1000G Phase 3 data repository. We then explored whether the observed taxonomic groups represent any previously described cryptic taxa within the *An. gambiae* complex. To do this, we constructed a NJT including Goundry, Tengrela (Crawford et al. 2016; Tennessen et al. 2021) and *Anopheles fontenillei* (Barrón et al. 2019) from West Africa. We also constructed a NJT with recognised members of the species complex including *An. merus*, *An. melas* and *An. quadriannulatus* (Fontaine et al. 2015). In addition, the *f*_3_ and *f*_4_ statistics as described by Patterson et al. (2012) were used to examine shared ancestry and assess admixture among the mosquito populations under study. Additionally, genetic diversity across species and population cohorts was assessed using *F*_ST_ analysis and the commonly used summary statistics nucleotide diversity (𝜃𝜋), Tajima's *D* and Watterson's theta (*θ*_*W*_). We also calculated Hudson's *F*_ST_ measure of genetic differentiation (Crawford et al. 2016; Tennessen et al. 2021) between population cohorts using the malariagen_data python package.

#### 2.3.2 Insecticide Resistance

SNPs and CNVs in known insecticide resistance genes including *Vgsc*, *Ace‐1*, *Rdl*, Gste, COE and cytochrome P450s were assessed to identify potentially functional mutations associated with insecticide resistance. Amino acid substitution frequencies across all population cohorts were computed, and those exceeding 5% in at least one cohort were retained. Frequencies of CNVs with copy numbers greater than two were calculated using methods described in Lucas et al. (2019). Genes of focus were those linked to metabolic insecticide resistance such as the *Cyp6aa/p*, *Cyp9k1*, *Cyp6m/z*, *Gste* and Coe gene clusters. Samples with high coverage variance exceeding 0.2 were excluded due to their impact on CNV calling reliability. We also performed hierarchical diplotype clustering using city block distance and complete linkage. To identify variants associated with low heterozygosity diplotypes likely under selection, heterozygosity, gene copy number and amino acid substitutions were plotted onto the dendrogram.

#### 2.3.3 Signals of Selection

To detect both hard and soft sweeps, the H_12_ statistic was used to determine the haplotype homozygosity across window segments of the genome, as described by Garud et al. (2015). Statistical peaks indicative of selective sweeps were identified by plotting H_12_ values for each population cohort. The window size was calibrated for each sample cohort to accommodate demographic variation. The optimal size of 3000 windows was determined by plotting H_12_ value distributions across a range of sizes and selecting the point where the 95th percentile was at or below 0.1.

To investigate whether haplotypes with a signal of selection are shared among populations, a new statistic based on Garud et al.'s H_1_ statistic (Garud et al. 2015), called H_1X_, was implemented. While H_1_ estimates the probability that two haplotypes sampled from the same population are identical, H_1X_ extends this estimation to comparing the probabilities between populations:

$$H_{1X}=\sum_{i=1,\ldots n}p_{i}q_{i}$$

where for a genome region with n distinct haplotypes, *p*_*i*_ is the frequency of the *i*th haplotype in the first population, and *q*_*i*_ is the frequency of the *i*th haplotype in the second cohort.

H_1X_ is a descriptive statistic and can be used to identify outlier genome regions where the degree of haplotype sharing between two populations is higher than the genome‐wide average. H_1X_ values were plotted over a window size of 1000 base pairs for each population cohort.

To investigate the spread of haplotypes carrying insecticide resistance substitutions, median‐joining networks as described in Bandelt et al. (1999) were constructed, using a maximum edge distance of two SNPs to group closely related haplotypes. The Hamming distance was also constructed based on all haplotype pairs, and pairwise genetic distance was used to perform single linkage hierarchical clustering. In this analysis, clusters with identical or closely related haplotypes suggest an increased frequency of the genetic backgrounds due to positive selection.

All analyses were conducted using the *malariagen_data* Python package (Miles et al. 2024).

## 3 Results

### 3.1 Taxonomic Structure

The *An. gambiae* complex comprises numerous morphologically indistinguishable species. Individuals that could not be assigned to known species have also been described from nearby East Africa (Crawford et al. 2016; Barrón et al. 2019; Tennessen et al. 2021). In this study, we therefore first sought to comprehensively investigate the species present in our data set and assess evidence for cryptic taxa. Our initial analysis used ancestry informative markers (AIMs) to make provisional assignment of *An. gambiae* complex species within our data set (see Figure S1). The majority of samples (*N* = 225) were provisionally assigned as *An. arabiensis* and 64 were assigned as *An. gambiae*. In addition, we found 11 individuals that could not be assigned to a known species within the complex (see Table 1). We did not identify any *An. coluzzii* samples in our dataset.

**TABLE 1:** Table summary showing the distribution of *Anopheles gambiae* complex mosquitoes collected in Tanzania at the respective locations and time (year).

Location	Collection year	Number of mosquitoes collected		
		*An. arabiensis*	*An. gambiae*	Unknown species
Moshi	2012	40	0	0
Tarime	2012	47	0	0
Muheza	2013	1	32	10
Muleba	2015	137	32	1

It is important to note that AIMs are based on a limited number of genomic markers identified from a restricted set of individuals—in this case, *An. arabiensis*, *An. gambiae* and *An. coluzzii*. Therefore, variation at these markers may not necessarily capture the full scope of genetic diversity across all our samples and may also fail to recognise or incorrectly assign individuals from previously unknown taxa. Consequently, principal component analysis (PCA) and neighbour‐joining trees (NJT) techniques were employed to further investigate the taxonomic structure of these samples.

**FIGURE 2:**
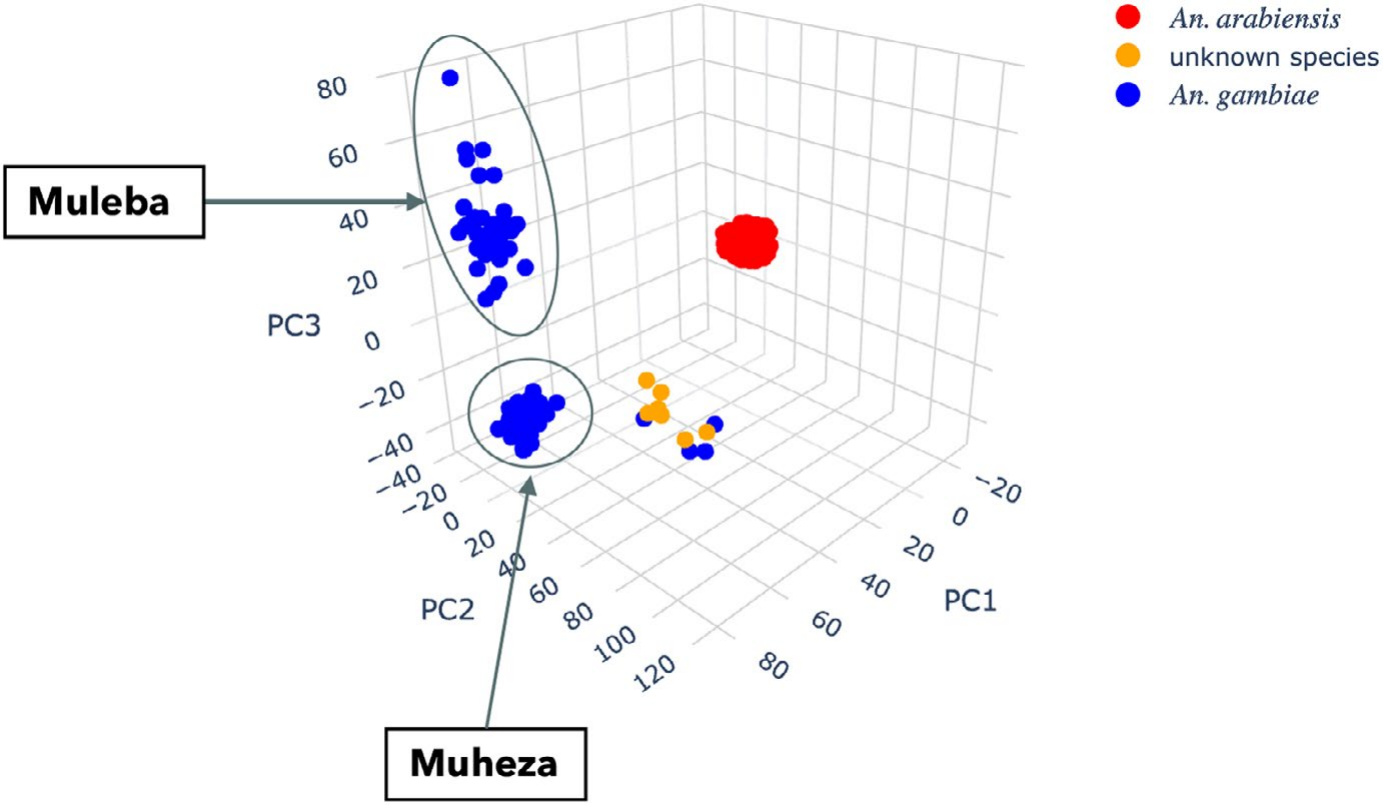
Principal component analysis (PCA) plot showing the population structure of *Anopheles gambiae* complex mosquitoes from Tanzania as provisionally assigned by AIMs, computed using SNPs from chromosome arm 3L.

**FIGURE 3:**
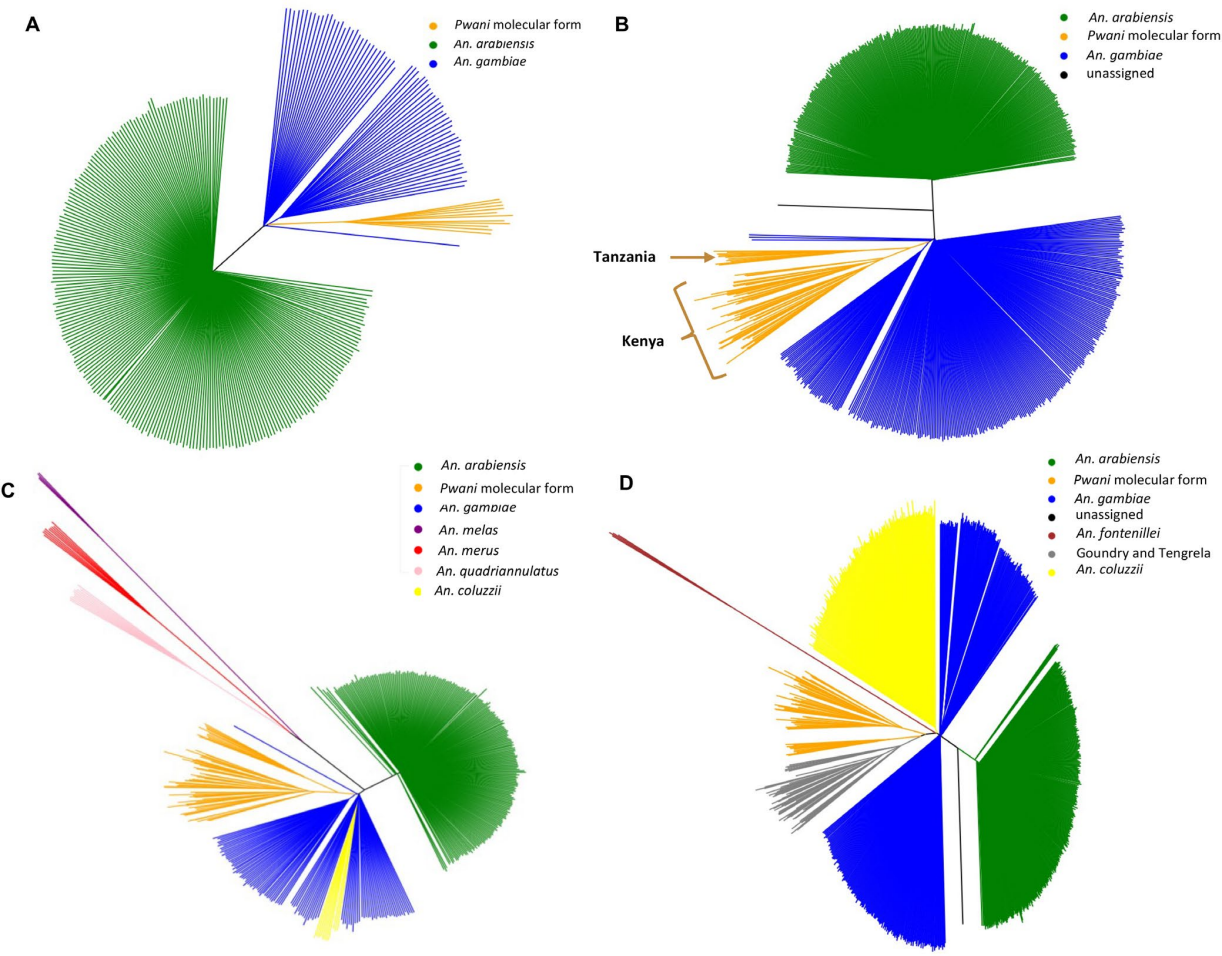
Neighbour Joining Trees (NJT) plots showing the population structure of *Anopheles gambiae*, *An. arabiensis*, and *Pwani* molecular form from (A) Tanzania, (B) Tanzania, Kenya and Uganda, (C) including *An. merus*, *An. melas* and *An. quadriannulatus*, and *An. coluzzii*, (D) including the most recent cryptic taxa such as *An. fontenillei*, Goundry and Tengrella from West Africa, computed using SNPs from chromosome arm 3L.

The PCA and NJT revealed four main clusters. Two of the clusters exclusively consisted of mosquitoes provisionally assigned from AIMs as *An. gambiae* from Muleba and Muheza, respectively. A third cluster comprised all mosquitoes provisionally assigned as *An. arabiensis* across the three locations. A fourth cluster largely consisted of individuals for whom species could not be assigned with AIMs (see Figures 2 and 3A) originating from Muheza. The fact that some individuals within this cluster were provisionally assigned as *An. gambiae* by the AIMS analysis highlights the increased resolution of genetic information obtained using SNPs widely distributed across a whole chromosome arm.

We then extended these analyses to include samples from neighbouring sites: Tororo and Kanungu in Uganda, approximately 588 and 414 km away from Muleba, respectively, and Kilifi, a coastal county in Kenya about 335 km from Muheza. The PCA (Figure S3) and NJT (Figure 3B) analysis revealed five clusters from which *An. arabiensis* across all countries clustered together, *An. gambiae* mosquitoes from Muleba clustered with those from the Ugandan sites, while those from Muheza clustered with those from Kenya. Additionally, two closely positioned clusters consisted of individuals which could not be assigned to known species, one cluster consisting of mosquitoes from the coast of Kenya (Kilifi) and another from the coast of Tanzania (Muheza) (see Figure 3B and Figure S3).

Previous research has documented the discovery of new cryptic taxa within the *An. gambiae* complex. Recent additions to this taxonomy, primarily identified in West Africa, include Goundry and Tengrela in Burkina Faso, as reported by Crawford et al. (2016) and Tennessen et al. (2021), respectively, and *Anopheles fontenillei* in Gabon, as documented by Barrón et al. (2019). Although an overlap between these newly discovered cryptic taxa in West Africa and individuals with unknown taxonomic status along the East African coast is highly unlikely due to geographical distance, further investigations were conducted to ascertain this potential overlap. Results from the NJT analysis unequivocally confirmed no overlap between these enigmatic individuals and the recently discovered cryptic taxa within the complex, namely Goundry, *An. fontenillei*, and Tengrela (see Figure 3C,D).

Subsequent investigation using supplementary data from Fontaine et al. (2015) was undertaken to ascertain whether individuals with unknown species status were associated with any of the presently recognised species within the *An. gambiae* complex, such as *An. merus*, *An. melas*, and *An. quadriannulatus*. Results from the NJT analysis ruled out the possibility that these individuals were either *An. merus*, *An. melas*, or *An. quadriannulatus* (see Figure 3C).

**FIGURE 4:**
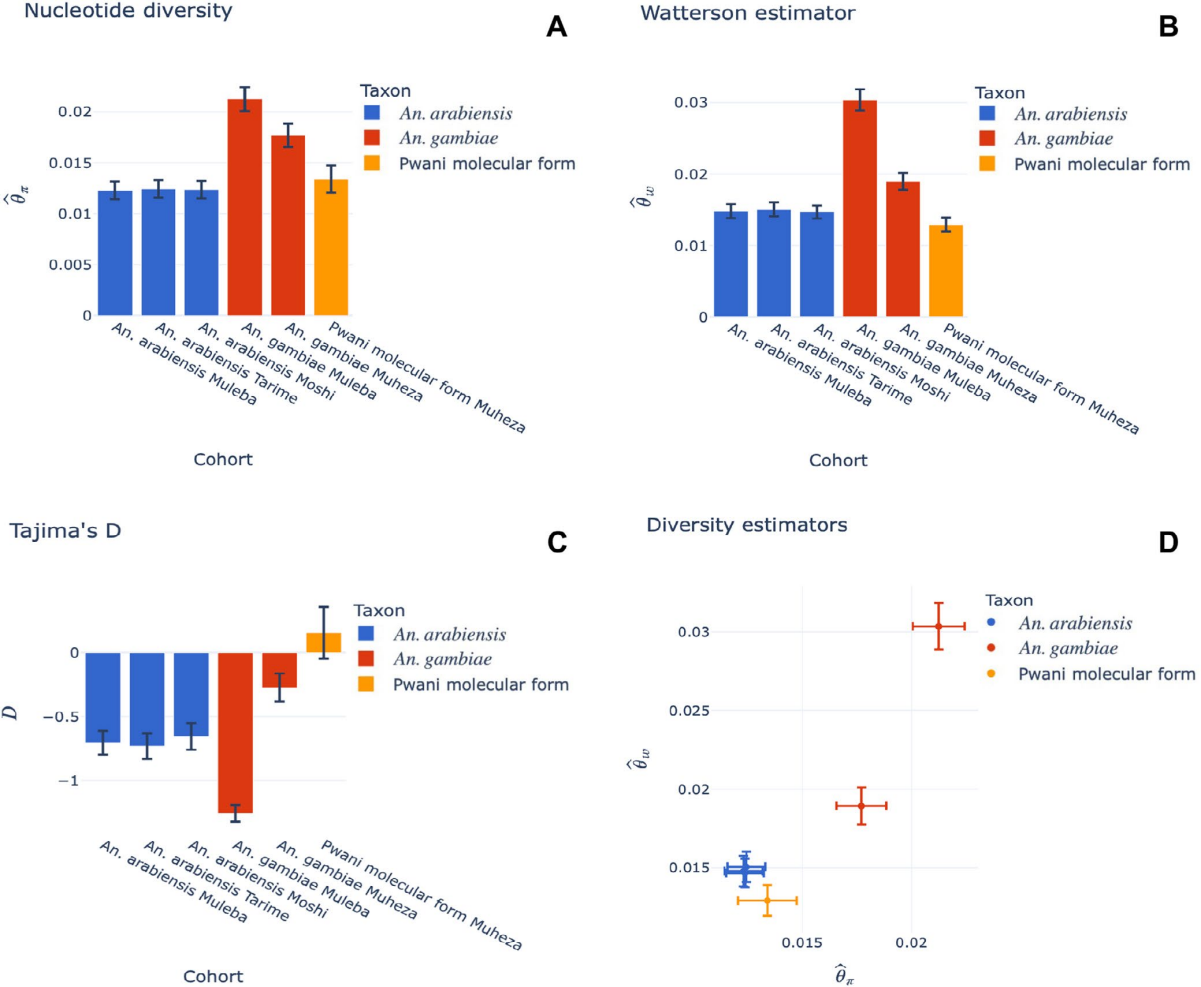
Genetic diversity summary statistics (A) Nucleotide diversity, (B) Watterson estimator, (C) Tajima's *D* and (D) Plot comparing all the estimators showing the diversity differentiation between different population cohorts of *Anopheles gambiae* complex mosquitoes collected in Tanzania.

To further confirm whether the individuals with an unknown taxonomic status were distinct from the known taxa within the complex, we conducted genetic diversity and *F*_ST_ analysis. The two groups with unknown taxonomic status have a positive Tajima's *D* value suggesting a population decline, while the *An. gambiae* s.s. and *An. arabiensis* populations have negative values, suggesting a population expansion (see Figure 4C). This suggests that the two populations with an unknown taxonomic status have a similar demographic history which is distinct from the known species. Similarly, a high genetic differentiation is observed between the two groups with unknown taxonomic status and *An. gambiae* s.s. For example, *F*_ST_ values ranged between 0.176 and 0.213 on comparison with *An. gambiae* population cohorts from Tanzania and Kenya. Similarly, *F*_ST_ was higher on comparison with *An. arabiensis* with values ranging from 0.344 to 0.353 (Figure S5). These *F*_ST_ values further suggest that the two groups with an unknown taxonomic status are distinct from the known species.

Further analysis was done to assess whether the two groups with an unknown taxonomic status are a result of hybridisation between any two known taxa within the complex, using the *f*_3_ statistic. The *f*_3_ statistic specifically tests whether one test population is a result of admixture or hybridisation between two reference populations (Patterson et al. 2012). Our analysis indicates no evidence of admixture/hybridisation between any of the known species within the complex, as all *f*_3_ values were positive (see Figure S6). Therefore, these findings suggest the plausible existence of a cryptic mosquito species within the *An. gambiae* complex along the coast of Kenya and Tanzania. This assertion is supported by evidence of their distinctive genetic structure, their unique profile of genetic diversity, and the absence of evidence that they are a result of hybridisation between any known species.

Subsequently, we assessed the potential of shared ancestry between the two groups of cryptic taxa from the coast of Kenya and Tanzania given their geographical proximity. PCA analysis revealed the clusters of these two groups cluster along the same principal component, consistent with shared ancestry (Figure S3). Furthermore, similar demographic characteristics, such as population decline based on diversity estimators, and a low *F*_ST_ value (0.005) further support this hypothesis. Moreover, Patterson's *D* (ABBA BABA) or *f*_4_ statistic further confirmed common ancestry between the two groups, as all the *f*_4_ statistic values were not significantly different from 0 (zero) when tested as sister branches in all reference comparisons (Figure S7). We propose to designate this new cryptic taxon as the *An. gambiae*, *Pwani* molecular form (‘*Pwani*’).

### 3.2 Geographical Structure

The population structure of the identified species collected from various regions across the country was analysed to assess evidence for geographical isolation or restricted gene flow. Principal Component Analysis (PCA) and Neighbour joining trees (NJTs) revealed two distinct clusters of *An*. gambiae. One cluster consisted of individuals from the western region (Muleba—Kagera), while the second cluster was composed exclusively of individuals from the eastern region (Muheza—Tanga) of Tanzania. This finding suggests that there is limited gene flow between the eastern and western parts of the country for this species. Conversely, *An. arabiensis* mosquitoes from all three collection sites clustered together in one group, suggesting extensive gene flow within the species as shown by the *F*_ST_ (Muleba‐Tarime *F*_ST_ = 0.000; Muleba‐Moshi *F*_ST_ = 0.001; Moshi‐Tarime *F*_ST_ = 0.002) (see Figure S5). In support of findings from the PCA, population cohorts of *An. arabiensis* from Muleba, Tarime, and Moshi exhibited similar values of nucleotide diversity, Watterson's estimator, and Tajima's *D* (Figure 4). Moreover, there were noticeable differences in values of all diversity estimators between *An. gambiae* populations from the western (Muleba) and eastern (Muheza) parts of Tanzania, which also displayed a relatively high degree of genetic differentiation (*F*_ST_ = 0.048) (see Figure S5). Lastly, the newly discovered *Pwani* molecular form was only observed at the coastal sites, that is, Muheza and Kilifi, suggesting that it might have a limited geographical range.

### 3.3 Insecticide Resistance

#### 3.3.1 Target Site Resistance

**FIGURE 5:**
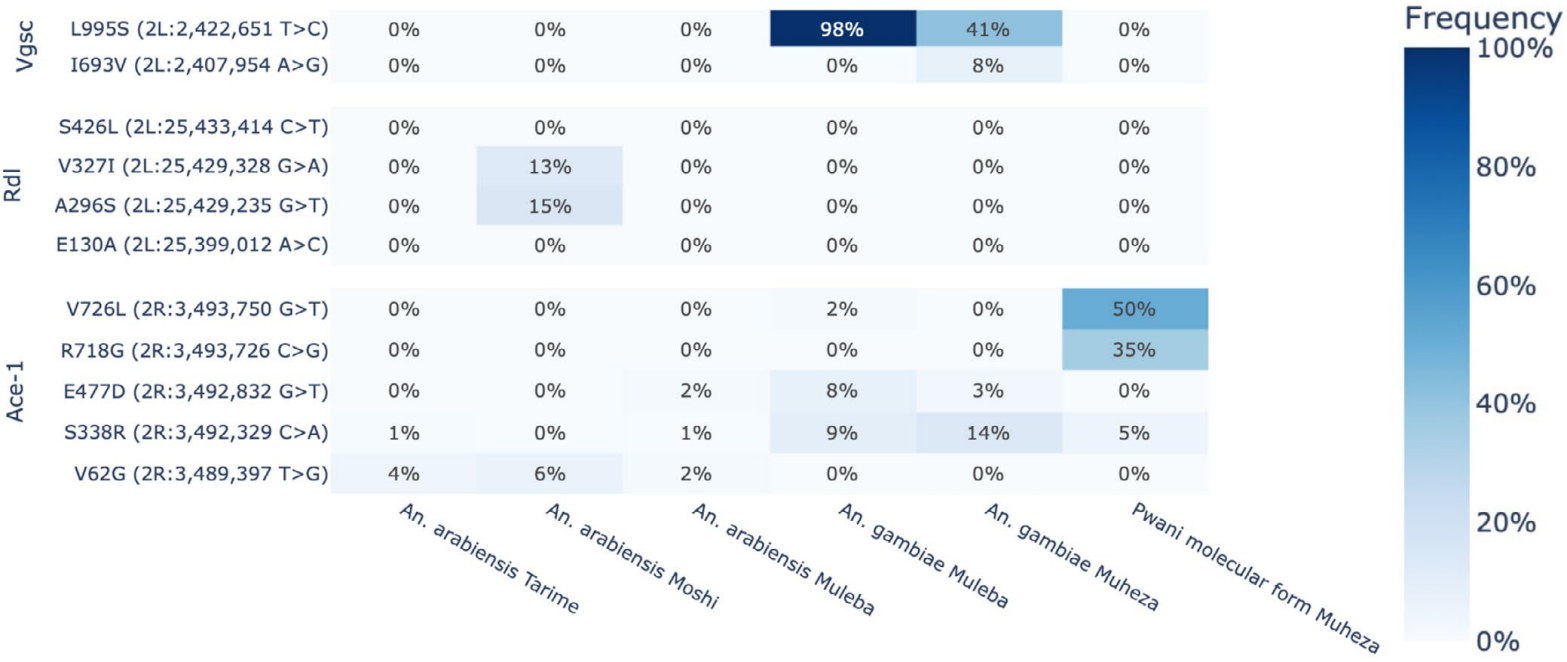
Heat map showing non‐synonymous amino acid substitutions within the pyrethroid insecticide target *Vgsc*; the target site of dieldrin known as *Rdl* and the *Ace‐1* organophosphate/carbamate target site in *An. gambiae* complex mosquitoes collected from selected sites in Tanzania.

To assess the potential impact of insecticide resistance on vector control efforts, known resistance variants were identified based on the mechanism and allele frequencies were calculated across the sampling sites. In *An. gambiae* populations, two mutations were identified in the *Vgsc* gene: the well‐characterised L995S (‘*kdr‐east*’, also known as L1014S according to *M. domestica* codon numbering) and a novel I693V mutation, which to our knowledge has not been previously reported in this species in Tanzania (Figure 5). The L995S mutation was found at different frequencies in the western and eastern populations of *An. gambiae* in Tanzania. The Muleba population had the highest frequency of 98% and the Muheza population had a moderate frequency of 41%. The I693V allele was exclusive to the *An. gambiae* population from Muheza (8%), but its role in insecticide resistance is unknown. Interestingly, these findings show that the *An. arabiensis* populations across all sites, along with the newly discovered *Pwani* molecular form from Muheza, did not exhibit any of the target site mutations associated with pyrethroid or DDT resistance.

Substitutions at the GABA receptor *Rdl* were only observed in the *An. arabiensis* population from Moshi (see Figure 5). The *Ace‐1* substitution G289S associated with resistance to carbamates and organophosphates was not observed in any cohort. However, the different taxa had markedly different substitution profiles at the locus, with the most common substitutions being V26G in *An. arabiensis*, S338R/E477D in *An. gambiae*, and V726L/R718G in the *Pwani* molecular form, though none are known to be associated with resistance at present.

#### 3.3.2 Metabolic Resistance

**FIGURE 6:**
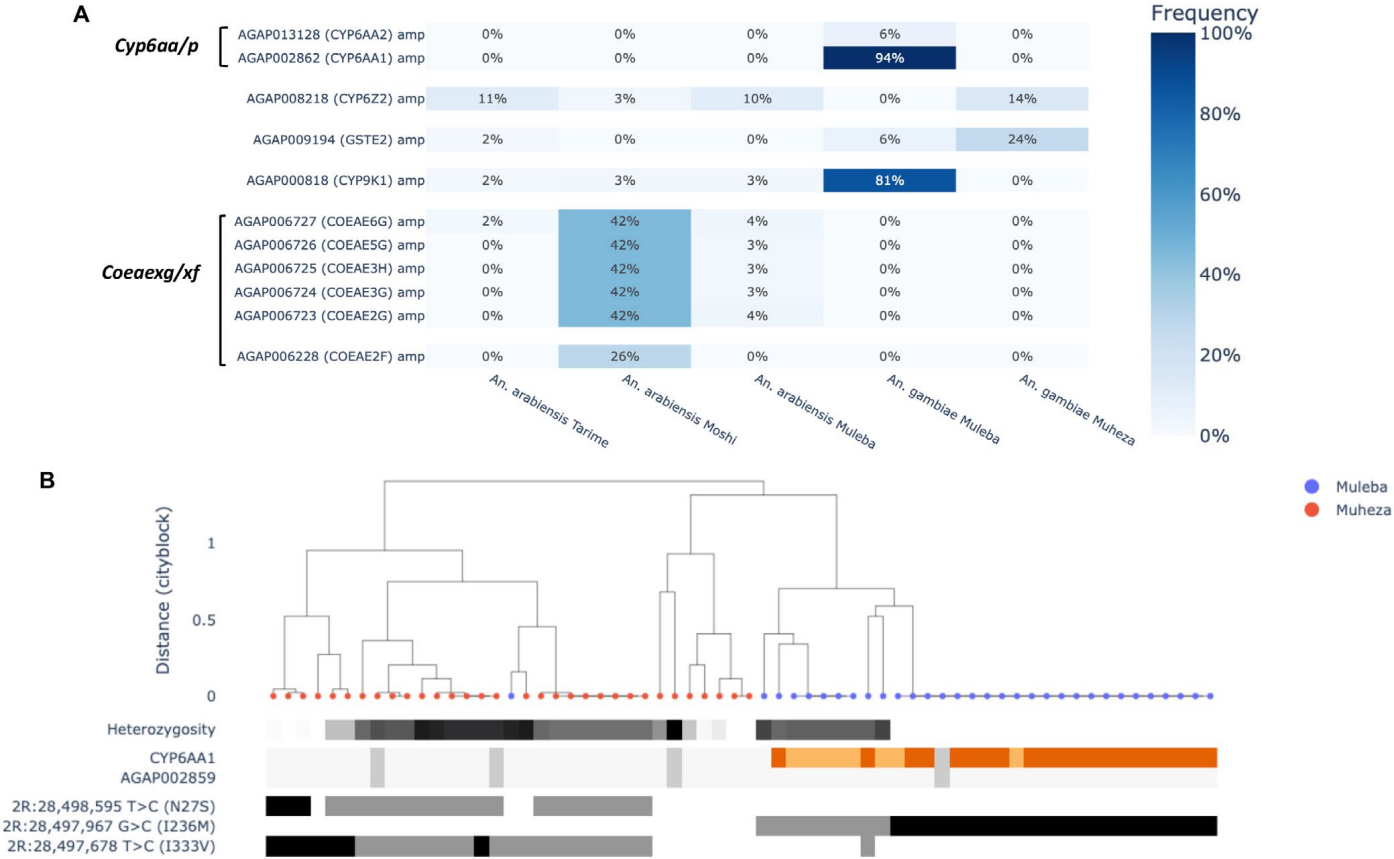
(A) Heat map showing copy number variation (CNV) frequencies of Cytochrome P450 and GSTe, and COE gene clusters associated with metabolic insecticide resistance (B). diplotype clustering in *Anopheles gambiae* complex mosquitoes collected from selected sites in Tanzania.

To investigate the presence of metabolic resistance driven by copy number variations (CNV), we computed the frequency of CNVs at associated cytochrome P450 genes on the *Cyp6aa/p* and *Cyp6m/z* clusters, *Cyp9k1*, *Coe* and *Gstu*‐*Gste* gene cluster for all the population cohorts (Figure 6A). Only the CNVs present in at least one cohort at a frequency of 5% or higher were included in the analysis. Overall, amplifications of cytochrome *P450* and *Gste* genes were more prevalent in *An. gambiae* compared to *An. arabiensis*. Specifically, *Cyp6aa/p* amplifications were absent from *An. arabiensis* while negligibly low frequency amplifications were detected at the *Cyp9k1* and *GSTe2* gene in *An. arabiensis* from Muleba, Moshi, and Tarime. However, we did observe *Coeae2f* and *Coeae2g–Coeae6g* amplifications in the *An. arabiensis* Moshi population at 26% and 42% frequencies, which were absent from *An. gambiae*.

The frequencies of CNVs in cytochrome P450 genes were also different between the northwestern and northeastern Tanzania sites. *Cyp6aa1*, a member of the *Cyp6* gene family, was the most frequently amplified gene at 94% and was found to be co‐occurring with *Cyp6aa2* at 6% in the *An. gambiae* population from Muleba but not in any of the other populations. Similarly, the *Cyp9k1* gene was also commonly amplified with a frequency of 81% (see Figure 6A). In contrast, *An. gambiae* from Muheza showed no evidence of CNV amplification at these genes except for 14% amplification of *Cyp6z2*. We also observed that the *An. gambiae* cohort from Muheza had a modestly higher 24% amplification of the *Gste2* gene compared to 6% in Muleba.

To investigate whether the cohort from Muleba could harbour the recently reported CNV associated with a triple mutant in East Africa known to cause high‐level insecticide resistance in *An. gambiae* as described by Njoroge et al. (2022), we investigated the discordant reads at the *Cyp6aa/p* cluster. *An. gambiae* from Muleba were found to have the previously described duplication known as Dup1, which forms part of the triple mutant along with a nonsynonymous point mutation in *Cyp6p4* (I236M). We further investigated whether this mutation was present in the population and found that the mutation I236M was common in *An. gambiae* from Muleba at an 83% frequency but absent from all other cohorts (Figure S12). We also used diplotype clustering to identify CNVs and candidate substitutions at the *Cyp6p4* region uniquely associated with clusters of low heterozygosity, which indicates they are under selection. We observed one diplotype cluster with low heterozygosity prevalent in Muleba. As expected, individuals within this cluster had high gene copy numbers across the *Cyp6aa/p* region and the I236M substitution associated with the duplication (Figure 6B).

Moreover, bioassay results were available for samples from Muheza, which were part of a larger population tested for susceptibility to deltamethrin (0.05%) using WHO bioassays. Among the total 948 samples tested, 22.3% were resistant to deltamethrin (Kabula et al. 2016). Samples from Tarime, Muleba and Moshi were not subjected to bioassays. However, the *An. arabiensis* samples from Moshi underwent microarray analysis targeting *Cyp6m2*, *Cyp6p3*, *Cyp4g16* and ABC 2060. The results revealed a high expression of *Cyp4g16* and ABC—transporter 2060 genes, suggesting a potential role in the observed pyrethroid resistance in this population (Matowo et al. 2014). Conversely, lower expression levels of *Cyp6m2* and *Cyp6p3* indicate that resistance mechanisms other than insecticide metabolism may be contributing to the observed pyrethroid resistance (Matowo et al. 2014).

### 3.4 Genome‐Wide Selection Scans

**FIGURE 7:**
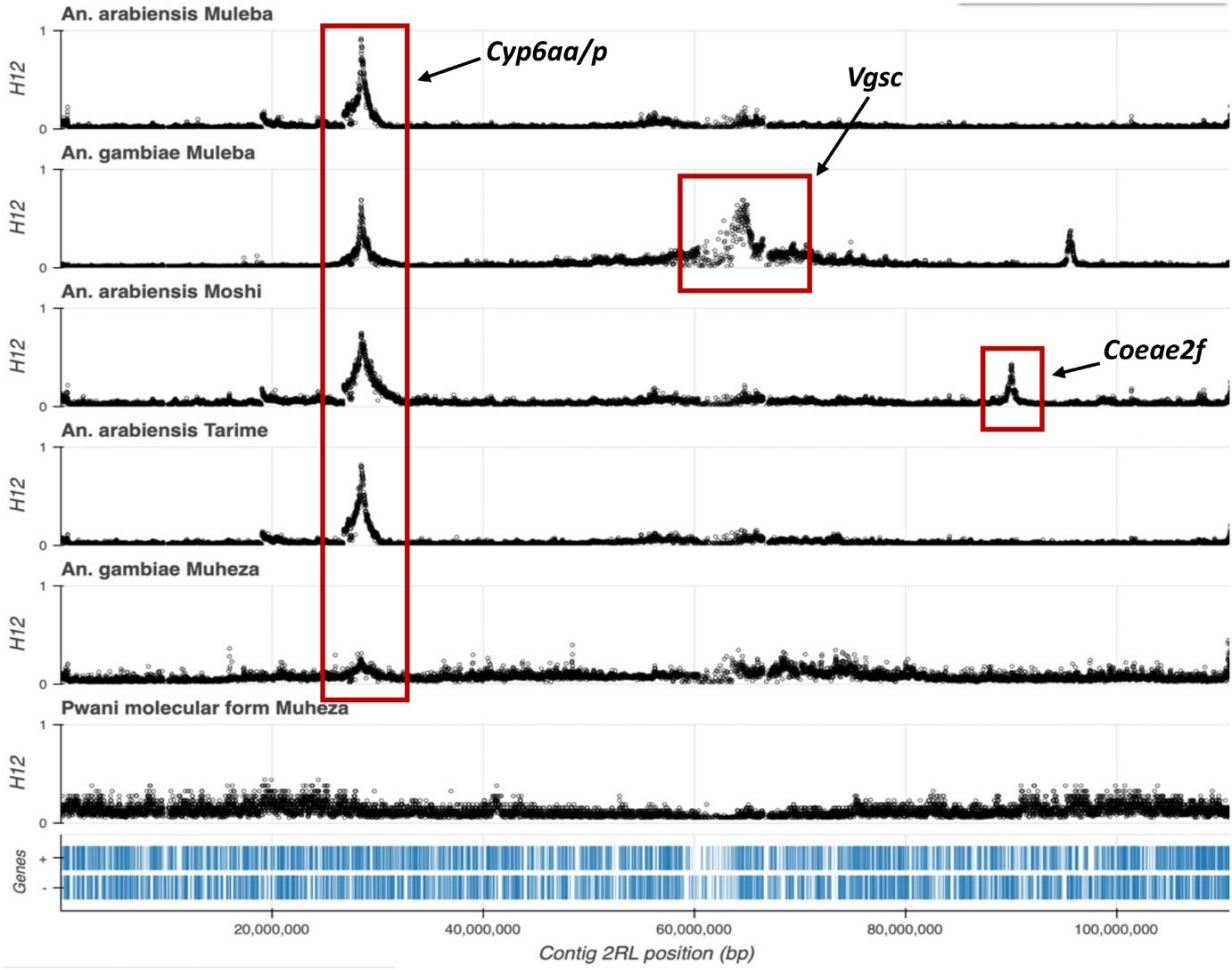
Genome‐wide selection scans (GWSS) showing selection sweeps labelled with the likely drivers of the respective selections on chromosome 2 of *Anopheles gambiae* complex mosquito populations collected across four sites in Tanzania.

To detect any presence of novel loci under selection in *An. gambiae* complex populations from Tanzania, we conducted genome‐wide selection scans (GWSS) using the H_12_ statistic. Our findings indicate a distinct peak in the vicinity of the established pyrethroid metabolic insecticide resistance (*Cyp6aa/p*) locus on the 2R chromosome arm at approximately 28.5 Mbp across all five population cohorts of *An. arabiensis* and *An. gambiae*, though the peak was less obvious in the *An. gambiae* population from Muheza (see Figure 7). These peaks suggest the genetic region is likely under positive selection in all these species except for the *Pwani* molecular form.

These findings are consistent with our observation of insecticide resistance associated SNP and CNVs in the *An. gambiae* population cohorts. However, this is not the case for *An. arabiensis*, which shows negligibly low frequencies of CNvs around the *Cyp6aa/p* loci where we observe a significant signal of selection in all populations. These results suggest that, even if insecticide resistance mutations are present at this locus in these populations, they may be driven by amino acid variations, indels or structural variants not captured by the CNV caller.

Furthermore, while we also observed moderate amplification of the *Coeae2f* and *Coeae2g‐Coeae6g* gene cluster in the *An. arabiensis* population from Moshi (see Figure 6A), no selection signal was observed in the *Coeae2g‐Coeae6g* gene cluster. However, there is evidence of a selection signal near the *Coeae2f* gene in the same population, which is consistent with the results obtained from the CNV analysis.

Moreover, on chromosome arm 2L, a selective peak at the *Vgsc* gene approximately at 28.5 Mbp in *An. gambiae* from Muleba was observed, which supports our finding of high frequencies of the L995F substitution associated with pyrethroid resistance in this population cohort. However, a clear selective peak was not seen at this region for any other cohort, although an indistinct signal was observed for *An. gambiae* from Muheza. In comparison to *An. gambiae* and *An. arabiensis*, we do not observe signals of selection in the *Pwani* molecular form. However, reduced genetic diversity within this group reduces the capacity to detect molecular signals. Additionally, no clear selective peaks were observed along either chromosome 3 or X for any population cohort.

### 3.5 Spread of Insecticide Resistance

**FIGURE 8:**
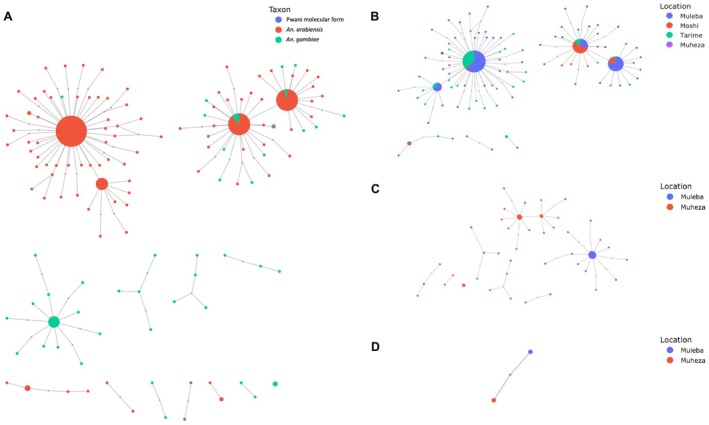
Haplotype networks showing the sharing of haplotypes at the *Cyp6aa/p* locus associated with metabolic resistance, across (A) All taxa, (B) *An. arabiensis*, (C) *An. gambiae* and (D) *Pwani* molecular form mosquitoes collected from four selected sites in Tanzania.

Since multiple selective peaks were observed at the 2R *Cyp6aa/p* locus across different species and geographical populations, the H_1X_ statistic was used to investigate signals of shared adaptive gene flow. The sharing of these haplotypes across species and geographies was also visualised using haplotype networks (Figure 8) and hierarchical clustering (see Figure S11). Within species, a common peak at the *Cyp6aap/p* locus was observed in *An. arabiensis* population cohorts from Muleba, Tarime and Moshi, indicating that haplotypes are shared between these cohorts (see Figure S9A). This finding is consistent with the lack of population structure observed between different geographical locations for this species, which also suggests a possible spread of insecticide resistance across geographies in this species (see Figure 8B).

In contrast, there was no evidence of adaptive gene flow between *An. gambiae* populations from the eastern and western sites, which is consistent with the observation of different CNV variants in these populations (see Figure S9B). This is also consistent with findings from our PCA and diversity analyses, which suggest restricted gene flow between *An. gambiae* populations from the eastern and western parts of Tanzania (see Figures 2 and 4). Similarly, the haplotype network also suggests that there has been no spread of insecticide resistance haplotypes between these populations (see Figure 8C).

The comparison between species was indicative of putative adaptive introgression at the *Cyp6z* locus between *An. gambiae* from Muheza and all populations of *An. arabiensis* from Muleba, Tarime and Moshi, but not with the Muleba population of *An. gambiae*, and we observe a peak between these species on the H_1X_ analysis (see Figure S10). These results indicate that insecticide resistance mutations are not only spreading within species but across species (see Figure 8A).

## 4 Discussion

This study used whole‐genome sequence data to investigate the population structure and genetic insecticide resistance profiles of the *An. gambiae* complex mosquitoes collected across four regions in northern Tanzania. Firstly, our results reveal the presence of a new genetically distinct subgroup found in sympatry with *An. gambiae* on the northeastern coast in Tanga, which shares common ancestry with a group found on the southeastern coast of Kenya in Kilifi, based on *f*_4_ and admixture analysis. Our analysis supports the conclusion that these two groups are part of a cryptic taxon within the *An. gambiae* complex, not previously described, and hereby named the *Pwani* molecular form. A number of cryptic species have been recently reported from West Africa including Goundry, Tengrela, *Anopheles fontenillei* (Tennessen et al. 2021; Crawford et al. 2016; Barrón et al. 2019). However, the *Pwani* molecular form is the newest cryptic taxon discovered within the complex in East Africa, following the earlier discoveries of *An. merus* in the 1950s and 60s (Thomson 1951; Paterson and World Health Organization 1962; Mattingly 1977), confined to the coast from South Africa to the Horn of Africa (Sattler et al. 2005); *An. bwambae* found in 1985 in the geothermal hot springs of Semliki Forest, Uganda (Mattingly 1977; White 1985); and the 2013 distinction between *Anopheles amharicus* in the Horn of Africa and *An. quadriannulatus* in east and southern Africa (Coetzee et al. 2013). This taxon exhibits genetic distinctiveness from known species, evidenced by its unique genetic diversity profile, separate clustering on PCA and NJT plots, high *F*_ST_ values compared to known species, and differences in insecticide resistance allele frequencies.

The discovery of the *Pwani* molecular form raises important questions about its origin, ecology, geographical range, susceptibility to insecticides, and ability to transmit malaria parasites, with unknown implications for malaria control. While its ecology and geographical range are not fully understood, the study suggests it may be restricted to the coast. *An. gambiae*, *An*. *merus* and *An. arabiensis* are currently considered the primary malaria vectors along the coast of Kenya and Tanzania (Bartilol et al. 2021). Initially, *An*. *merus*, which so far is also considered to be geographically confined to the coast, was thought to play a minor role in malaria transmission (Mutero et al. 1984). However, recent studies have elevated its vector status in certain coastal regions such as the coast of Kenya and Tanzania (Temu et al. 1998). During dry seasons, *An. merus* densities peak as freshwater species decline, potentially sustaining malaria transmission along the coast (Bartilol et al. 2021). Similarly, while the contribution of the *Pwani* molecular form to malaria transmission is not yet fully understood, its geographical confinement and potential adaptation to saltwater habitats suggest it could pose a similar threat.

Results also show that the *Pwani* molecular form has a positive Tajima's *D* statistic indicating a recent population bottleneck, while population expansion was evident in *An. gambiae* and *An. arabiensis*. A prior study reported that the population decline observed in the peculiar *An. gambiae* form from coastal Kenya, which is found to also be the *Pwani* molecular form in this study, was not due to the introduction of ITNs in the area. This is because ITNs were introduced 6 years prior to the sample collections in the study site, whereas the inferred decline occurred approximately 200 generations (~5000 years) before the date of sampling (Miles et al. 2017). This decline could be due to historical environmental events or prolonged human activities that predate the introduction of ITNs at these sites, such as drought or climatic changes. Further research may be needed to identify and understand the specific causes behind the population decline in this species.

Interestingly, we did not observe any mutations associated with insecticide resistance or signals of selection at either the target site or metabolic resistance genes in the *Pwani* molecular form, which contrasts with observations in *An. gambiae* and *An. arabiensis*. The taxon may have a distinct ecology and behaviour, potentially allowing it to evade contact with insecticide‐based vector control tools; although the samples were collected indoors where contact with IRS and ITN products is likely. Alternatively, it may be that the *Pwani* molecular form has a small effective population size and therefore a low likelihood of resistance emergence and spread.

Analysis of the geographical structure of *An. gambiae* complex mosquitoes revealed notable differences between *An. arabiensis* and *An. gambiae*. As for *An. arabiensis*, results show that populations from Tanzania (Muleba, Tarime and Moshi), Uganda (Tororo and Kanungu) and Kenya (Kilifi) exhibit the same genetic diversity and cluster together in PCA and NJT plots, indicating a lack of population differentiation and high gene flow among these populations. This aligns with previous studies that reported a lack of strong population structure and high gene flow on a regional scale for *An. arabiensis* populations (Nyanjom et al. 2003; Besansky et al. 1997), except for island populations experiencing historical drifts (Simard et al. 1999). The lack of population substructure in this species may be attributed to its physiological and behavioural plasticity, as it is highly adaptable and has a greater tolerance for drier environments (Ayala and Coluzzi 2005; Donnelly et al. 1999). Therefore, despite regional climatic variations within the country, this species' adaptability to various climatic environments can support their dispersal, survival and reproduction across Tanzania and the neighbouring countries. The uniform population structure among different populations of *An. arabiensis* across geographical borders may facilitate adaptive advantage, including the spread of mutations conferring insecticide resistance within the species population.

Contrary to the observations found in *An. arabiensis*, a pronounced geographical population structure was observed between *An. gambiae* in the northwestern and northeastern parts of Tanzania. Extended analysis, including samples from Uganda and Kenya, showed that Ugandan samples from Tororo and Kanungu had a similar genetic composition and clustered together with *An. gambiae* from Muleba in western Tanzania, while samples from Kilifi on the eastern coast of Kenya clustered with those from Muheza in eastern Tanzania (see Figure S3). These results suggest restricted gene flow between eastern and western *An. gambiae* populations within East Africa, likely due to the Great Rift Valley acting as a physical barrier which has been previously implicated in promoting population substructure in this species (Ng'habi et al. 2011). Moreover, the Rift Valley passes through the central corridor of the country which includes regions with semi‐desert and desert conditions which are unsuitable for *An. gambiae* species which prefer a humid climate. The findings in this study coincide with those of previous studies which have reported that *An. gambiae* populations tend to be more structured than *An. arabiensis* due to physiological and ecological differences (Ng'habi et al. 2011; Ayala and Coluzzi 2005; Donnelly et al. 1999), which explains the difference in population structure observed in this study between these two species.

Overall, these findings imply that targeted interventions may enhance malaria control in Tanzania, particularly for *An. gambiae* due to its restricted gene flow and varied genetic diversity, necessitating region‐specific control strategies. This approach will be useful as it would allow for more effective use of resources and potentially higher success rates in reducing malaria transmission by addressing the unique characteristics of local mosquito populations. Conversely, a broader approach would be required for controlling *An. arabiensis* as it lacks population differentiation across various sites. These results may also inform new innovations, particularly the prospects and potential future implementation of genetically modified mosquitoes (GMM). For example, for *An. gambiae*, multiple releases may be needed to accommodate genetic differences, whereas for *An. arabiensis*, which appears to have much less geo‐isolation, multiple releases may not be necessary.

Furthermore, the population structure of species plays a crucial role in shaping the development and spread of insecticide resistance, as genetic connectivity between populations can accelerate the dissemination of resistance‐conferring mutations. As previously discussed, *An. arabiensis* populations in Tanzania generally exhibit similar genetic diversity, suggesting a high gene flow, including genes conferring insecticide resistance, which may result in a uniform insecticide resistance profile. However, despite the apparent high likelihood of high gene flow among this species populations, we observed moderate frequencies of V327I and A296S mutations in the *Rdl* gene, present in the Moshi population. These mutations have been associated with resistance to dieldrin in *An. funestus* from West and East Africa (Odero et al. 2024), and A296S specifically confers the same in *An. arabiensis*, *An. stephensi*, and *Aedes aegypti* (Du et al. 2005; ffrench‐Constant et al. 2000). Interestingly, a previous study by Mahande et al. (2012) also reported the presence of *Rdl* in the *An. arabiensis* population in lower Moshi, suggesting that insecticide residues from agricultural pesticides might contribute to the development of this resistance allele, given that malaria control in this area exclusively relies on pyrethroids impregnated in LLINs. The occurrence of *Rdl* mutations in *An. arabiensis* in lower Moshi is supported by other previous studies by Kishimba et al. (2004) and Hellar and Kishimba (2005) both highlighting the largest sugar estate in Tanzania—Tanganyika Planting Company Limited (TPC) sugarcane plantation in the Kilimanjaro region (in lower Moshi) as a location with high levels of residual insecticides due to intensive historical pesticide use of dieldrin and lindane (also known as technical grade HCH). However, the latter study also reports higher levels of technical HCH (β‐HCH) which strongly suggest a recent use of this chemical pesticide in the environment, which was banned In Tanzania by the Tropical Pesticides Research Institute in 2002 (Hellar and Kishimba 2005). In this case, the presence of this mutation in the Moshi population may be a result of long‐term pollution from stockpiles or areas where historical use was particularly intense, resulting in residual pollution that maintains selection pressure for *Rdl* in the population. It may also be an alarming indication of ongoing illegal use of these chemical pesticides in agricultural activities. Therefore, there is a need to continually monitor eco‐toxic environmental pollutants, minimise their levels and educate farmers on the impact of illegal use of these banned pesticides.

The *An. arabiensis* populations from Moshi were also distinct because they had moderate amplifications of the *Coeae2f* and *Coeae2g‐Coeae6g* genes as well as mutations associated with dieldrin resistance. The amplification of *Coeae2f* in this population is further supported by signals of selection near these gene's loci. Previous studies have reported that the two alpha‐esterases *Coeae1f/Coeae2f* are responsible for enhancing the ability of the *Culex pipiens* house mosquitoes to detoxify organophosphate insecticides, which were commonly used in larviciding campaigns (Guillemaud et al. 1997). More recently, orthologs of these genes, together with an amplification of the *Ace‐1* gene, have been observed in *An. gambiae* from West Africa and have been associated with the detoxification of the organophosphate pirimiphos‐methyl (formulated as Actellic CS300) (Nagi et al. 2024), which is widely used in IRS campaigns in sub‐Saharan Africa.

Contrary to *An. arabiensis*, the pronounced population substructure between the eastern and western *An. gambiae* populations suggests that there is a restricted gene flow, potentially leading to different resistance profiles in these populations. In this study, two mutations were identified at the *Vgsc* gene with varying frequencies between the two *An. gambiae* populations. Firstly, the known L995S (‘*kdr*‐East’) at 98% in Muleba and 41% in Muheza populations. This mutation, first discovered in the same species in western Kenya, has also been associated with permethrin and DDT resistance in various insect species, including *An. gambiae* from West Africa (Ranson et al. 2000).

Several mutations and signals of selection were also observed in the *An. gambiae* species at cytochrome P450 genes particularly amplified in the Muleba population, including *Cyp6aa1* (98%), *Cyp9k1* (81%), and *Cyp6aa2* (6%). The amplification of the *Cyp6aa1* gene is associated with the metabolism of deltamethrin in *An. coluzzii* (Lucas et al. 2023) and with the metabolism of pyrethroids, carbamates (dioxacarb) and bendiocarb used in IRS in *An. funestus* (Ibrahim et al. 2018), indicating potential implication of cross‐resistance in this and other *Anopheline* species. *Cyp9k1* has been associated with the metabolism of deltamethrin and pyriproxyfen in *An. gambiae* from Equatorial Guinea (Vontas et al. 2018) and is overexpressed in DDT and pyrethroid‐resistant strains of the same species in Cameroon (Fossog Tene et al. 2013). *Cyp6aa2* was reported to be associated with deltamethrin resistance in *An. minimus* mosquitoes from Thailand (Rongnoparut et al. 2003). The accumulation of genome variation associated with *kdr* and metabolic resistance in the Muleba population of *An. gambiae* suggests pyrethroid resistance and potential cross‐resistance to other insecticide classes like carbamates. Similarly, the presence of Dup1, which forms part of the triple mutant along with a nonsynonymous point mutation in *Cyp6p4* (I236M) in this population provides strong evidence of high resistance potential in this population. High selection pressure for pyrethroid resistance in Muleba is likely given that the district received special attention due to its high malaria prevalence and recurrent epidemics and thus received yearly rounds of IRS and LLINS with pyrethroids because of its high malaria prevalence (Kisinza et al. 2017). The area may benefit from urgent intervention of synergists or insecticide rotations. Moreover, *An. gambiae* from northeastern and northwestern Tanzania have different insecticide resistance profiles, which calls for a more targeted approach in the deployment of vector control interventions.

Lastly, while understanding within‐species population structure is crucial for tracking the geographical spread of adaptive variants, it is also important to monitor the cross‐species spread of such variants. For instance, the H_1X_ statistical analysis revealed a common peak at the *Cyp6aap/p* locus in *An. arabiensis* population cohorts from Muleba, Tarime and Moshi, as well as in *An. gambiae* from Muheza, suggesting shared haplotype adaptive introgression among these populations (see Figure S10A). These results show the complexity of managing resistances in malaria vectors across species and geographies.

## 5 Conclusion

The analysis of whole‐genome sequenced data from *An. gambiae* complex mosquitoes collected across northern Tanzania facilitated an in‐depth taxonomic analysis resulting in the discovery of a previously unidentified, cryptic taxonomic group, named the *Pwani* molecular form, apparently restricted to the coastal area of East Africa. Furthermore, we were able to determine species population structure with high resolution and with operational value to malaria control. The study revealed that *An. gambiae* from northwestern Tanzania displayed a geographical separation from the northeast with genomic evidence for stronger *kdr* and metabolic resistance to pyrethroids in comparison. In contrast, *An. arabiensis* exhibited consistent gene flow and resistance levels across regions. These contrasting findings underscore the importance of genomic analysis in identifying population structure and insecticide resistance mechanisms. However, the limited sample size underscores the need for additional research to determine the prevalence of the newly proposed taxon in these localities, as well as its population structure and continued occurrence in the area. Moreover, comprehensive data on its behaviour and vector competence will be crucial to establish its potential role in malaria transmission. We strongly emphasise that routine genomic surveillance is essential, offering valuable insights to policymakers and researchers, and in this case also provides strong evidence on the need to integrate a multisectoral approach in tackling malaria control and public health at large.

## Author Contributions

S.H.M. and K.L.B. analysed the data and wrote the manuscript. B.K. and J.M. contributed to fieldwork and data generation. S.A.B. and F.B. reviewed this manuscript, whereas S.C.N. contributed to data analysis. D.W., M.J.D., C.S.C., F.O.O. and A.M. contributed to conceptualisation, data analysis and writing.

## Disclosure

Benefits Generated: A collaboration was developed between the authors, including members from the country from which samples for genome sequencing originated. The partnership included knowledge transfer, with mentorship and training provided. This collaboration forms part of the MalariaGEN Vector Observatory, which is a global collaboration to generate an open access data resource for the genomic surveillance of Anopheles vectors of importance to public health. The results of research have been shared with the provider communities and the broader scientific community.

## Ethics Statement

The authors have nothing to report.

## Consent

The authors have nothing to report.

## Conflicts of Interest

The authors declare no conflicts of interest.

## Supporting information


Figure S1.
Figure S2.
Figure S3.
Figure S4.
Figure S5.
Figure S6.
Figure S7.
Figure S8.
Figure S9.
Figure S10.
Figure S11.
Figure S12.

## Data Availability

All data presented in the manuscript are available open source through the malariagen_data python package with source code available at https://github.com/malariagen/malariagen‐data‐python. The sequencing data generated in this study have also been submitted to the European Nucleotide Archive (ENA) under accession numbers ERR1554823‐ERR2532783.
