# Supplementary material for: Genomic Analysis Reveals a New Cryptic Taxon Within the Anopheles gambiae Complex With a Distinct Insecticide Resistance Profile in the Coast of East Africa
Source: Mol Ecol. 2025 Apr 16;34(10):e17762. doi: 10.1111/mec.17762 (PMC12051790; doi:10.1111/mec.17762)
Supplement: Supplementary file 1 — Figure S1. Figure S2. Figure S3. Figure S4. Figure S5. Figure S6. Figure S7. Figure S8. Figure S9. Figure S10. Figure S11. Figure S12. [file MEC-34-e17762-s001.zip › mec17762-sup-0012-FigureS11.pdf]

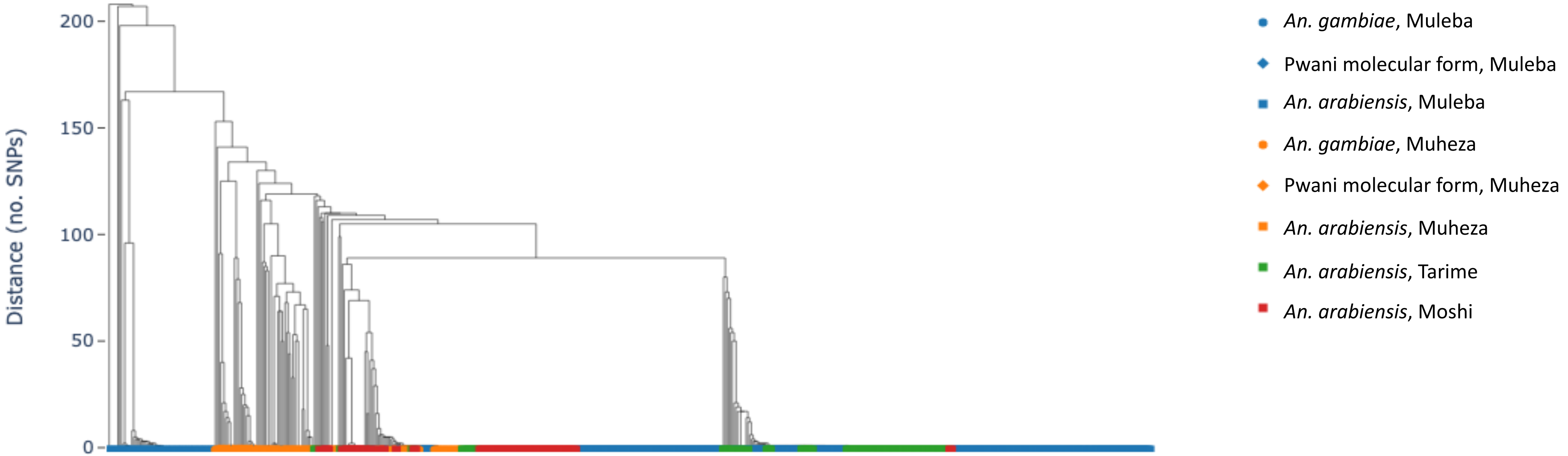

Distance (no. SNPs)
200
150
100
50
0
An. gambiae, Muleba
Pwani molecular form, Muleba
An. arabiensis, Muleba
An. gambiae, Muheza
Pwani molecular form, Muheza
An. arabiensis, Muheza
An. arabiensis, Tarime
An. arabiensis, Moshi
