# Supplementary material for: Genomic Analysis Reveals a New Cryptic Taxon Within the Anopheles gambiae Complex With a Distinct Insecticide Resistance Profile in the Coast of East Africa
Source: Mol Ecol. 2025 Apr 16;34(10):e17762. doi: 10.1111/mec.17762 (PMC12051790; doi:10.1111/mec.17762)
Supplement: Supplementary file 1 — Figure S1. Figure S2. Figure S3. Figure S4. Figure S5. Figure S6. Figure S7. Figure S8. Figure S9. Figure S10. Figure S11. Figure S12. [file MEC-34-e17762-s001.zip › mec17762-sup-0001-supinfo.docx]

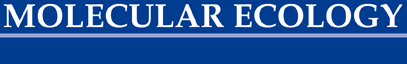


**Supplemental Information for:**

**Genomic analysis reveals a new cryptic taxon within the *Anopheles gambiae* complex with a distinct insecticide resistance profile in the coast of East Africa**

Sophia H. Mwinyi ^1, 2*+^, Kelly L. Bennett ^3^, Sanjay C. Nagi ^4^, Bilali Kabula ^5^, Johnson Matowo ^6^, David Weetman ^4^, Francesco Baldini ^2^, Simon A. Babayan ^2^, Martin J. Donnelly ^3, 4^, Chris S. Clarkson ^3^, Fredros O. Okumu ^1, 2, 7+^, Alistair Miles ^3+^

1. Environmental Health and Ecological Sciences Department, Ifakara Health Institute, P. O. Box 53, Ifakara, Morogoro, Tanzania.
2. School of Biodiversity, One Health and Veterinary Medicine, University of Glasgow, Glasgow, G12 8QQ, UK.
3. Genomic Surveillance Unit, Wellcome Sanger Institute, Wellcome Genome Campus, Hinxton, Cambridge, CB10 15A, UK.
4. Department of Vector Biology, Liverpool School of Tropical Medicine, UK.
5. National Institute for Medical Research (NIMR), Amani Centre, Muheza, Tanzania.
6. Kilimanjaro Christian Medical University College (KCMUCo), Tumaini University, Makumira, Moshi, Tanzania.
7. School of Life Science and Biotechnology, Nelson Mandela African Institution of Science and Technology, P.O. Box 447, Arusha, Tanzania.

**Table of Contents:**

| **Fig** | **Title** | **Page** |
| --- | --- | --- |
| 1 | Ancestry Informative Markers distinguishing *An. arabiensis* from *An. gambiae* & *An. coluzzii* and *An. gambiae* from *An. coluzzii* | 3 |
| 2 | Explained Variance of Principal Components Analysis of *An. gambiae* complex | 4 |
| 3 | Principal Component Analysis (PCA) Plot of *An. gambiae* complex mosquitoes from Tanzania, Kenya and Uganda | 5 |
| 4 | Genetic diversity summary statistics of *An. gambiae* complex populations  from Tanzania, Kenya and Uganda | 6 |
| 5 | Pairwise Genetic Differentiation (F_st_) Among *An. gambiae* complex  Populations from Tanzania and Kenya | 7 |
| 6 | *f₃*-statistic results showing status of admixture in unknown *An. gambiae*  complex taxa from Tanzania and Kenya | 8 |
| 7 | *f₄-*statistic results showing the status of shared genetic ancestry of unknown  *An. gambiae* complex Taxa from Tanzania and Kenya | 9 |
| 8 | Genome-Wide Selection Scans (GWSS) Identifying selection sweeps in  *An. gambiae* complex populations from Tanzania | 10 |
| 9 | H1X plots showing spatial adaptive gene flow at the *Cyp6aa/p* loci | 11 |
| 10 | H1X plots showing adaptive introgression gene flow at the *Cyp6aa/p* loci | 12 |
| 11 | Haplotype clusters showing the spread of resistance haplotypes along the  *Cyp6aa/p* loci in *An. gambiae* complex mosquitoes from Tanzania | 13 |
| 12 | Heat Map of Non-Synonymous Discordant Reads at Metabolic Resistance Genes in *An. gambiae* Complex Mosquitoes from Tanzania | 14 |

**Supplementary 1:** Ancestry Informative Markers plot showing a distinction between **A.** *An. arabiensis* and *An. gambiae* & *An. coluzzii*, **B.** *An. gambiae* and *An. coluzzii*

**
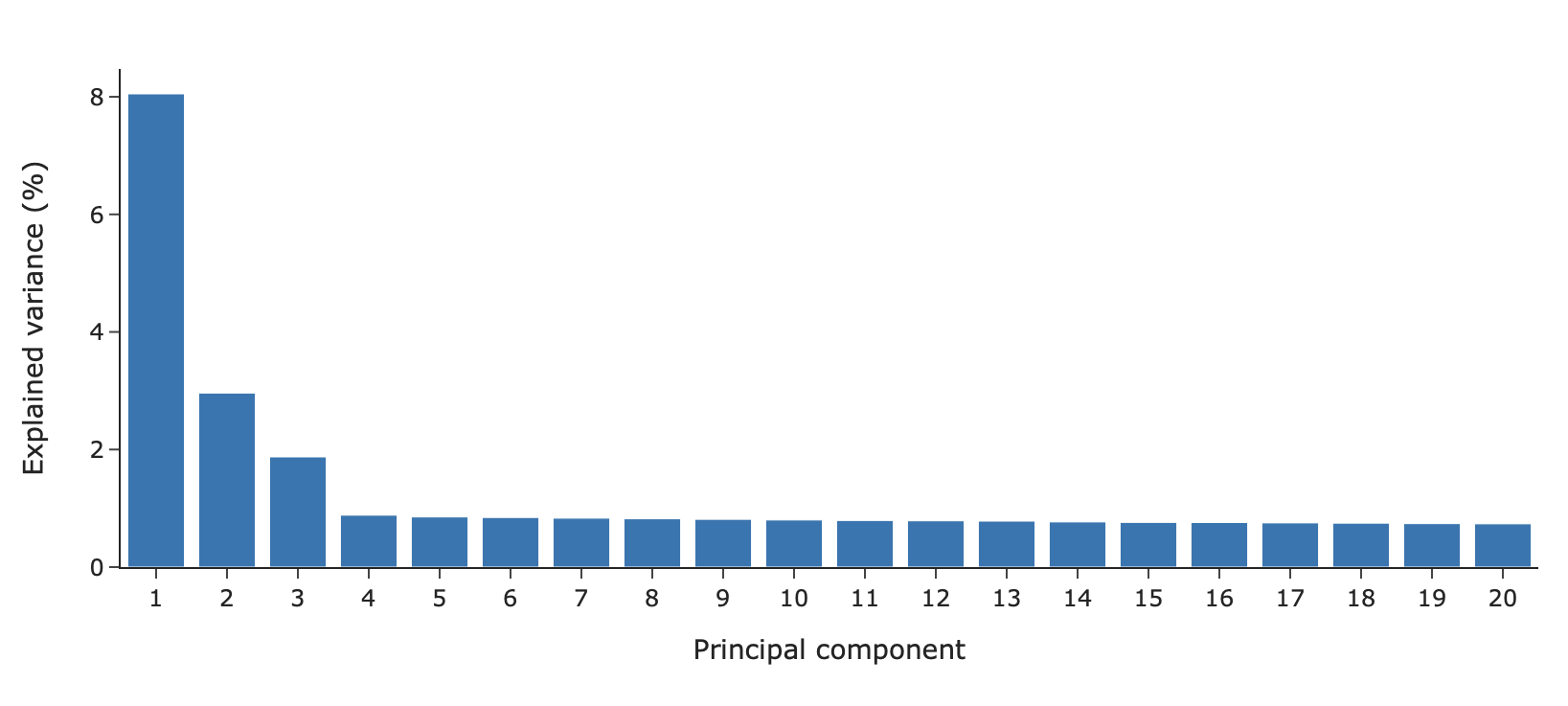
Supplementary 2:** Explained variance of the principal components analysis of the population structure of *An. gambiae* complex collected from four selected sites located in Northern Tanzania.

**Supplementary 3:** Principal component analysis (PCA) plot showing the population structure of *An. gambiae* complex mosquitoes from Tanzania, Kenya and Uganda, computed using SNPs from chromosome arm 3L.

**Supplementary 4:** Genetic diversity summary statistics **A**: Nucleotide diversity, **B**: Watterson estimator and **C**: Tajima’s D, and **D**: All Diversity Estimators showing the showing the genetic diversity between *An. gambiae* complex populations collected in Tanzania, Kenya and Uganda.

**Supplementary 5:** Pairwise genetic differentiation (***F_st_***) between *An. gambiae* complex populations from different districts in Tanzania and Kenya. ****

| ***Source 1*** | ***Source 2*** | ***Target*** | ***f_3_*** | ***SE*** | ***Z-score*** |
| --- | --- | --- | --- | --- | --- |
| ***Tanzania*** | | | | | |
| *An. gambiae* (TZ) | *An. arabiensis* (TZ) | *Pwani* molecular form (TZ) | 0.098 | 0.003 | 29.576 |
| *An. gambiae* (TZ) | *An. coluzzii* (CAR) | *Pwani* molecular form (TZ) | 0.079 | 0.003 | 28.478 |
| *An. gambiae* (TZ) | *An. melas* (Fontaine) | *Pwani* molecular form (TZ) | 0.084 | 0.003 | 28.343 |
| *An. gambiae* (TZ) | *An. quadriannulatus* (Fontaine) | *Pwani* molecular form (TZ) | 0.086 | 0.003 | 29.776 |
| *An. gambiae* (TZ) | *An. gambiae* (KE) | *Pwani* molecular form (TZ) | 0.083 | 0.002 | 39.227 |
| *An. gambiae* (TZ) | *An. arabiensis* (KE) | *Pwani* molecular form (TZ) | 0.098 | 0.003 | 29.601 |
| *An. gambiae* (TZ) | *An. coluzzii* (CAR) | *Pwani* molecular form (TZ) | 0.102 | 0.004 | 24.908 |
| *An. gambiae* (TZ) | *An. merus* (Fontaine) | *Pwani* molecular form (TZ) | 0.144 | 0.005 | 29.482 |
| *An. gambiae* (TZ) | *An. melas* (Fontaine) | *Pwani* molecular form (TZ) | 0.139 | 0.005 | 29.091 |
| *An. gambiae* (TZ) | *An. quadriannulatus* (Fontaine) | *Pwani* molecular form (TZ) | 0.143 | 0.005 | 30.745 |
| *An. gambiae* (TZ) | *An. gambiae* (KE) | *Pwani* molecular form (TZ) | 0.082 | 0.003 | 28.746 |
| *An. gambiae* (TZ) | *An. arabiensis* (KE) | *Pwani* molecular form (TZ) | 0.229 | 0.003 | 45.958 |
| ***Kenya*** | | | | | |
| *An. gambiae* (KE) | *An. arabiensis* (TZ) | *Pwani* molecular form (KE) | 0.358 | 0.007 | 49.611 |
| *An. gambiae* (KE) | *An. gambiae* (TZ) | *Pwani* molecular form (KE) | 0.219 | 0.006 | 33.859 |
| *An. gambiae* (KE) | *An. gambiae* (TZ) | *Pwani* molecular form (KE) | 0.216 | 0.006 | 36.440 |
| *An. gambiae* (KE) | *An. coluzzii* (CAR) | *Pwani* molecular form (KE) | 0.200 | 0.006 | 32.503 |
| *An. gambiae* (KE) | *An. merus* (Fontaine) | *Pwani* molecular form (KE) | 0.200 | 0.006 | 31.865 |
| *An. gambiae* (KE) | *An. melas* (Fontaine) | *Pwani* molecular form (KE) | 0.198 | 0.006 | 30.888 |
| *An. gambiae* (KE) | *An. quadriannulatus* (Fontaine) | *Pwani* molecular form (KE) | 0.201 | 0.006 | 32.210 |
| *An. gambiae* (KE) | *An. coluzzii* (CAR) | *Pwani* molecular form (KE) | 0.225 | 0.006 | 36.685 |
| *An. gambiae* (KE) | *An. merus* (Fontaine) | *Pwani* molecular form (KE) | 0.265 | 0.007 | 37.757 |
| *An. gambiae* (KE) | *An. melas* (Fontaine) | *Pwani* molecular form (KE) | 0.261 | 0.007 | 38.944 |
| *An. gambiae* (KE) | *An. quadriannulatus* (Fontaine) | *Pwani* molecular form (KE) | 0.266 | 0.008 | 34.528 |

**Supplementary 6:**  Table showing the ***f_3_* -** statistic results to determine whether the unknown taxa from Tanzania and Kenya are a result of admixture between the known taxa within *An. gambiae* complex

| ***Source 1 (A)*** | ***Source 2 (B)*** | ***Test (C)*** | ***Outgroup*** | ***f_4_*** | ***SE*** | ***Z-score*** |
| --- | --- | --- | --- | --- | --- | --- |
| *Pwani* molecular form (TZ) | *Pwani* molecular form (KE) | *An. arabiensis* (TZ) | *An. quadriannulatus* | 0.022 | 0.006 | 3.806 |
| *Pwani* molecular form (TZ) | *Pwani* molecular form (KE) | *An. arabiensis* (KE) | *An. quadriannulatus* | 0.013 | 0.006 | 2.203 |
| *Pwani* molecular form (TZ) | *Pwani* molecular form (KE) | *An. gambiae* (TZ) | *An. quadriannulatus* | 0.018 | 0.004 | 4.730 |
| *Pwani* molecular form (TZ) | *Pwani* molecular form (KE) | *An. gambiae* (KE) | *An. quadriannulatus* | 0.018 | 0.004 | 4.244 |
| *Pwani* molecular form (TZ) | *Pwani* molecular form (KE) | *An. merus* (Fontaine) | *An. quadriannulatus* | (0.004) | 0.007 | (0.530) |
| *Pwani* molecular form (TZ) | *Pwani* molecular form (KE) | *An. melas* (Fontaine) | *An. quadriannulatus* | 0.006 | 0.007 | 0.852 |
| *Pwani* molecular form (TZ) | *Pwani* molecular form (KE) | *An. coluzzii* (CAR) | *An. quadriannulatus* | (0.030) | 0.004 | (7.039) |

**Supplementary 7: *f_4_*-** statistic results to determine whether the unknown taxa from Tanzania and Kenya share a common genetic ancestry.

**Supplementary 8:** Genome-wide selection scans (GWSS) showing selection sweeps on **A**: Chromosome 3 and **B**: X chromosome, of *An. gambiae* complex mosquito populations collected from Muleba, Moshi, Tarime and Muheza in Tanzania.


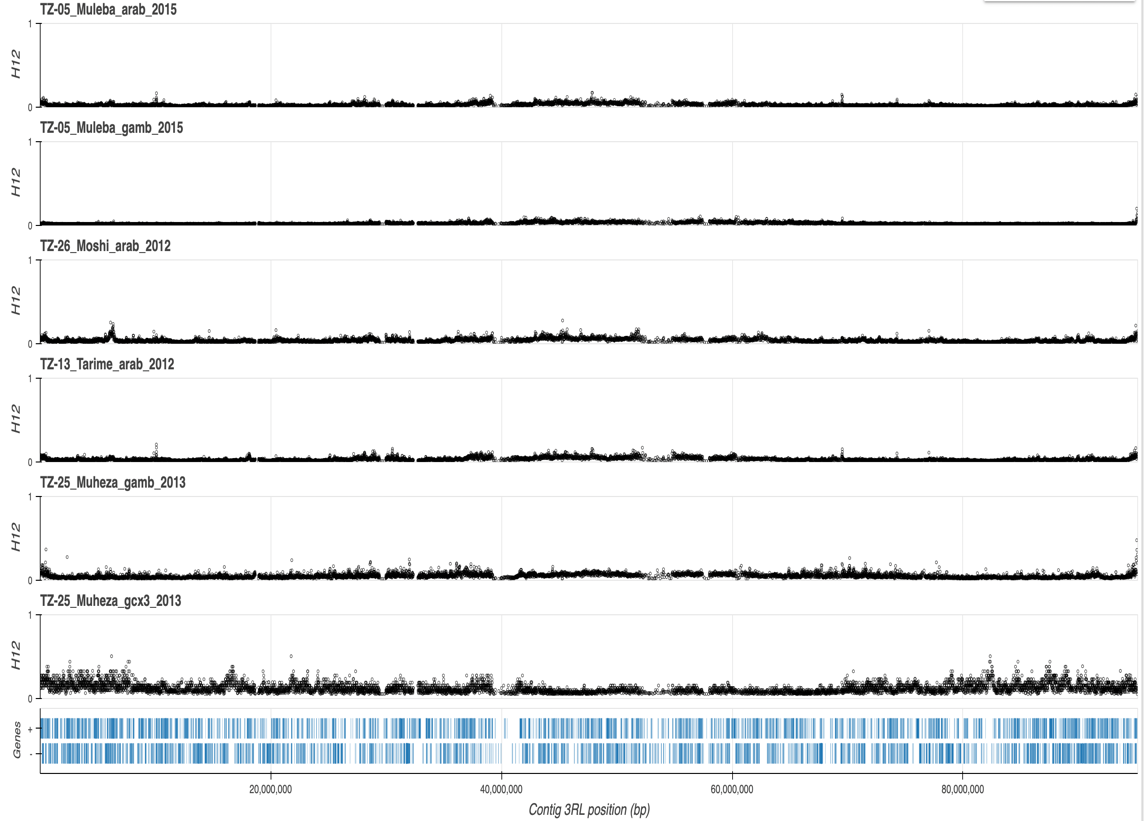

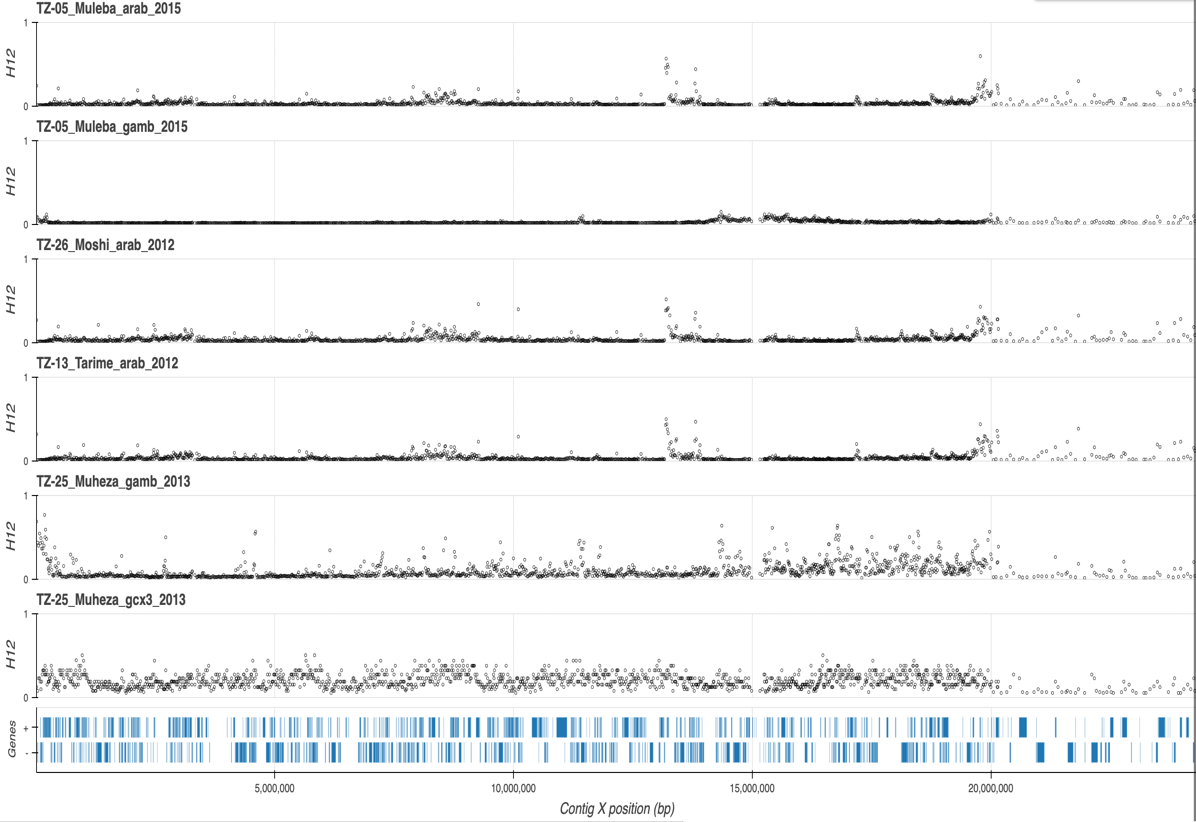


**A**

**B**


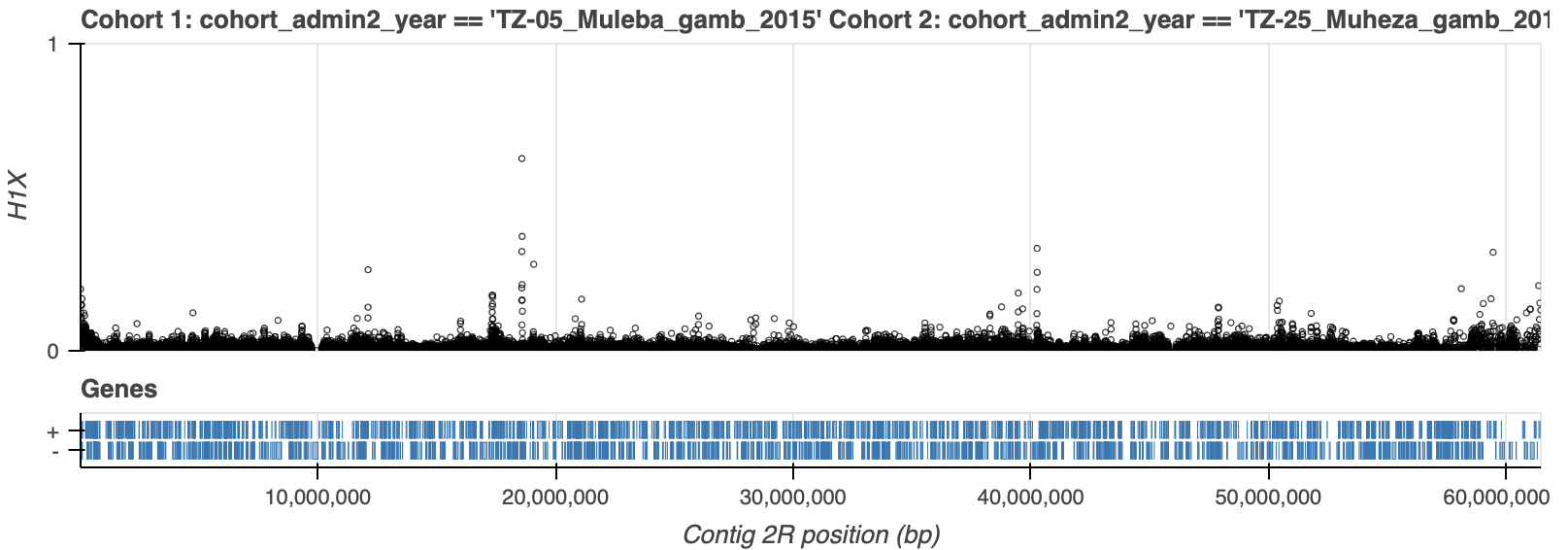

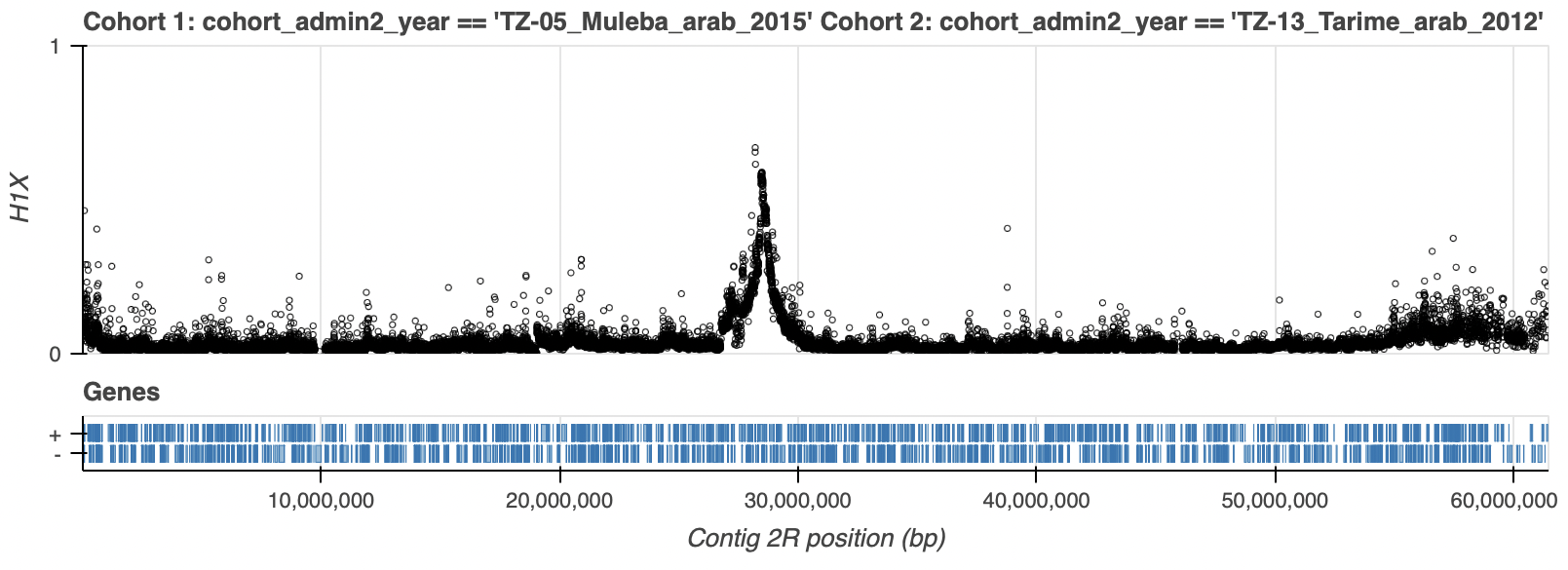

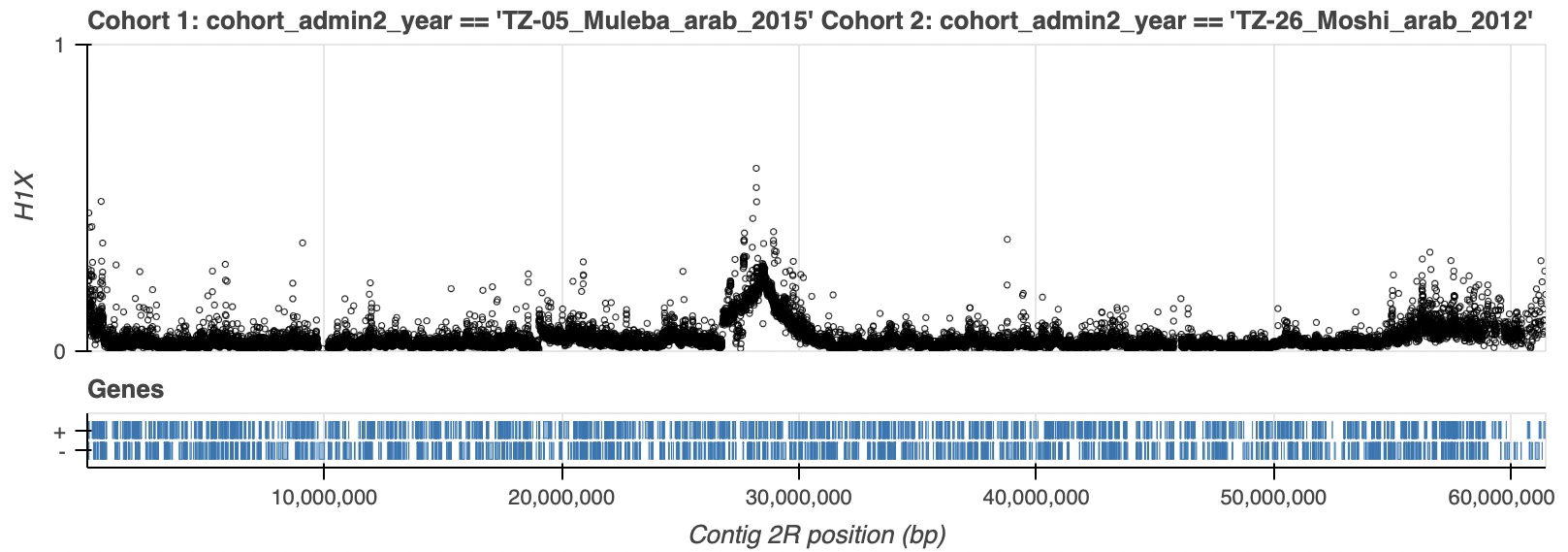

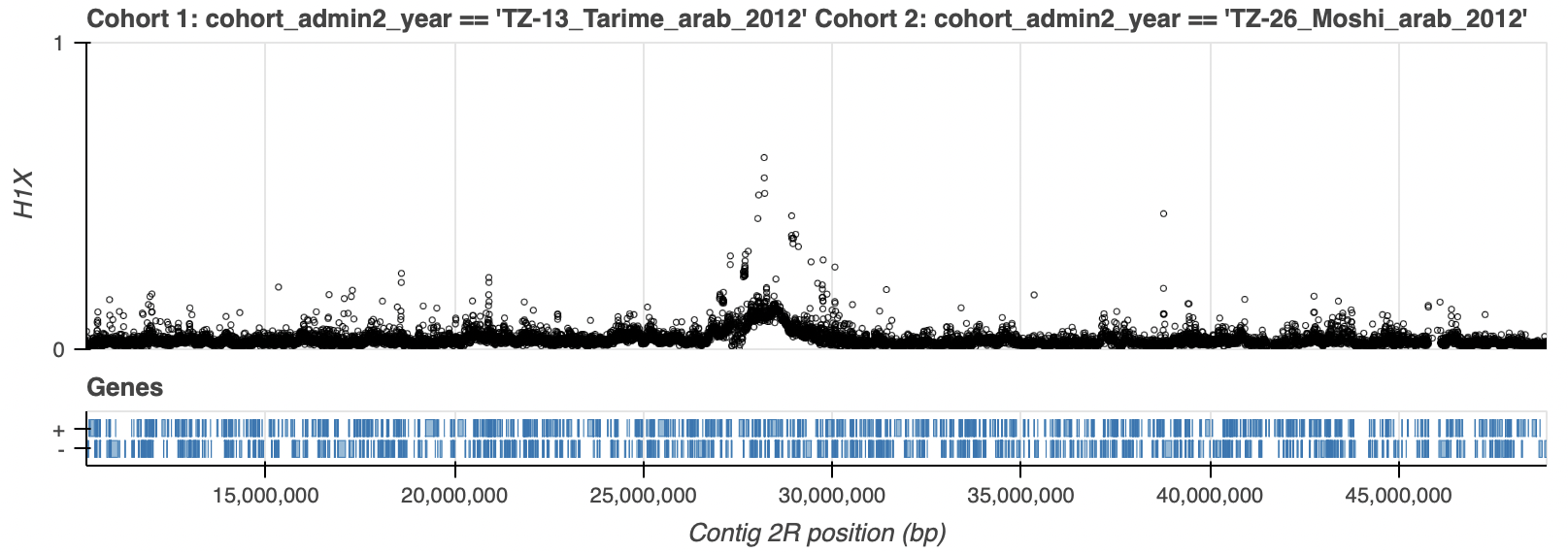


*An. arabiensis* cohorts

*An. gambiae* cohorts

**A**

**B**

**Supplementary 9:** H_1X_ plots showing spatial adaptive gene flow at the *Cyp6aa/p* loci between **A:** *An. arabiensis* population cohorts from Muleba, Moshi and Tarime and **B:** *An. gambiae* population cohorts collected from Muleba and Muheza in Tanzania.

**Supplementary 10:** H_1X_ plots showing adaptive introgression gene flow at the *Cyp6aa/p* loci between **A:** *An. arabiensis* population cohorts from Muleba, Moshi and Tarime and *An. gambiae* from Muheza, and **B:** *An. arabiensis* population cohorts from Muleba, Moshi and Tarime and *An. gambiae* from Muheza.


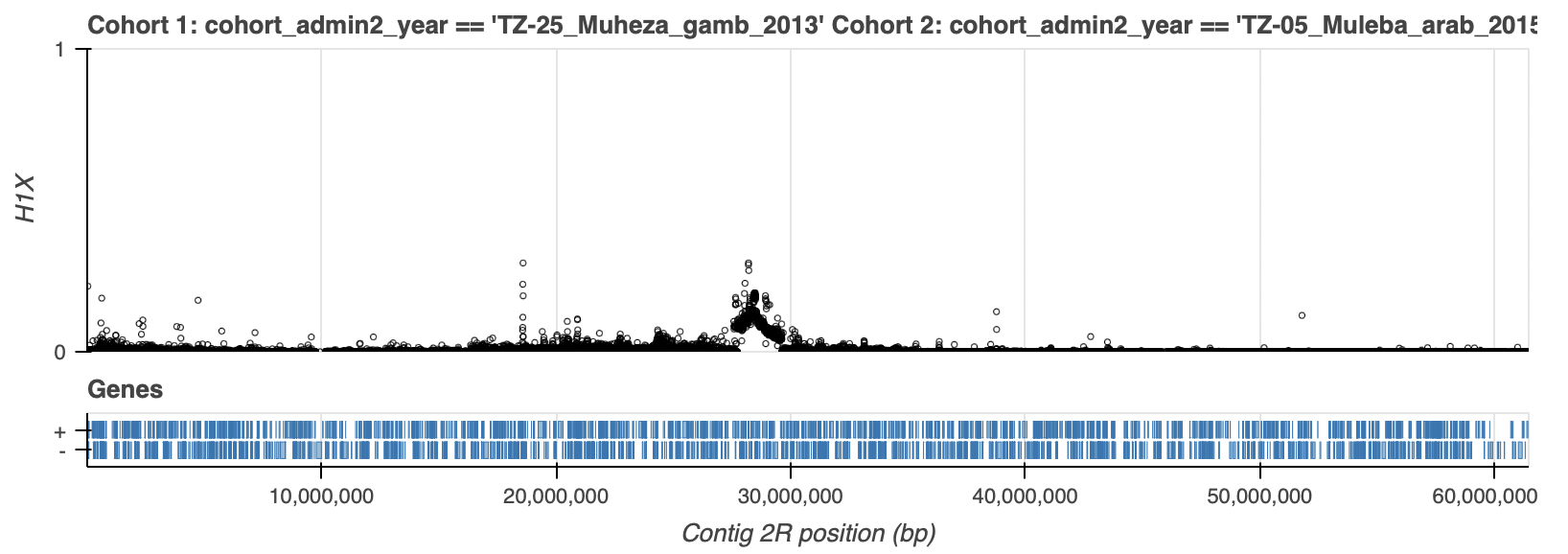

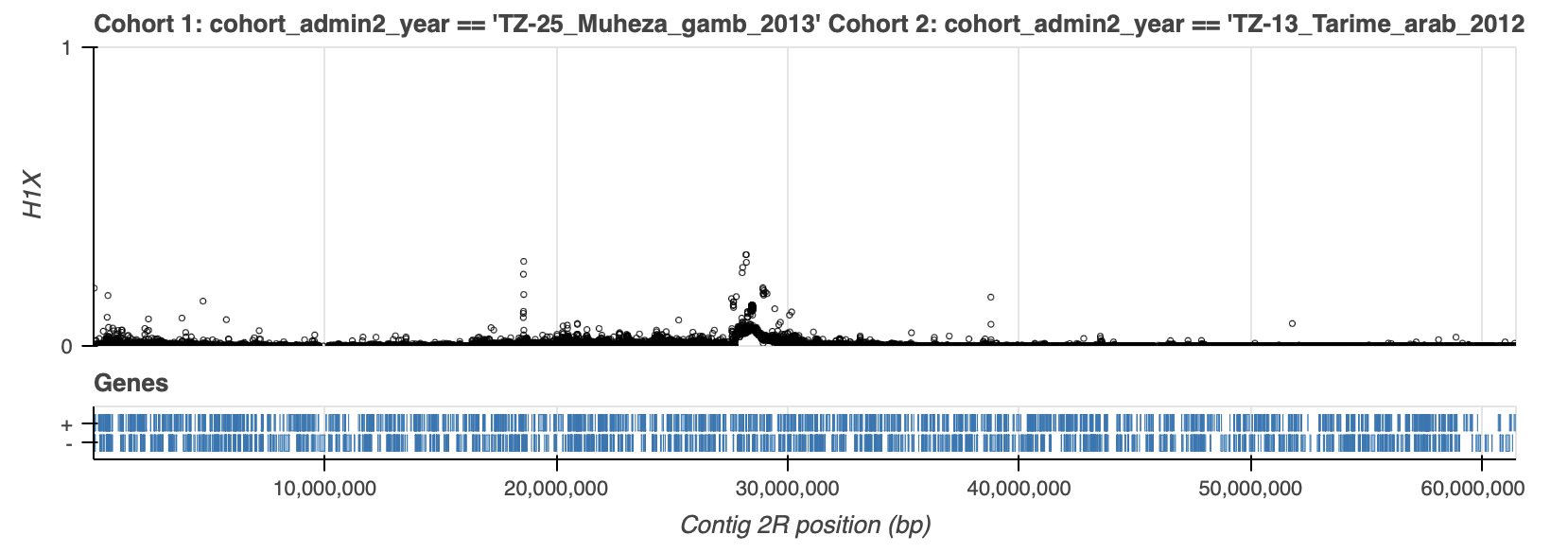

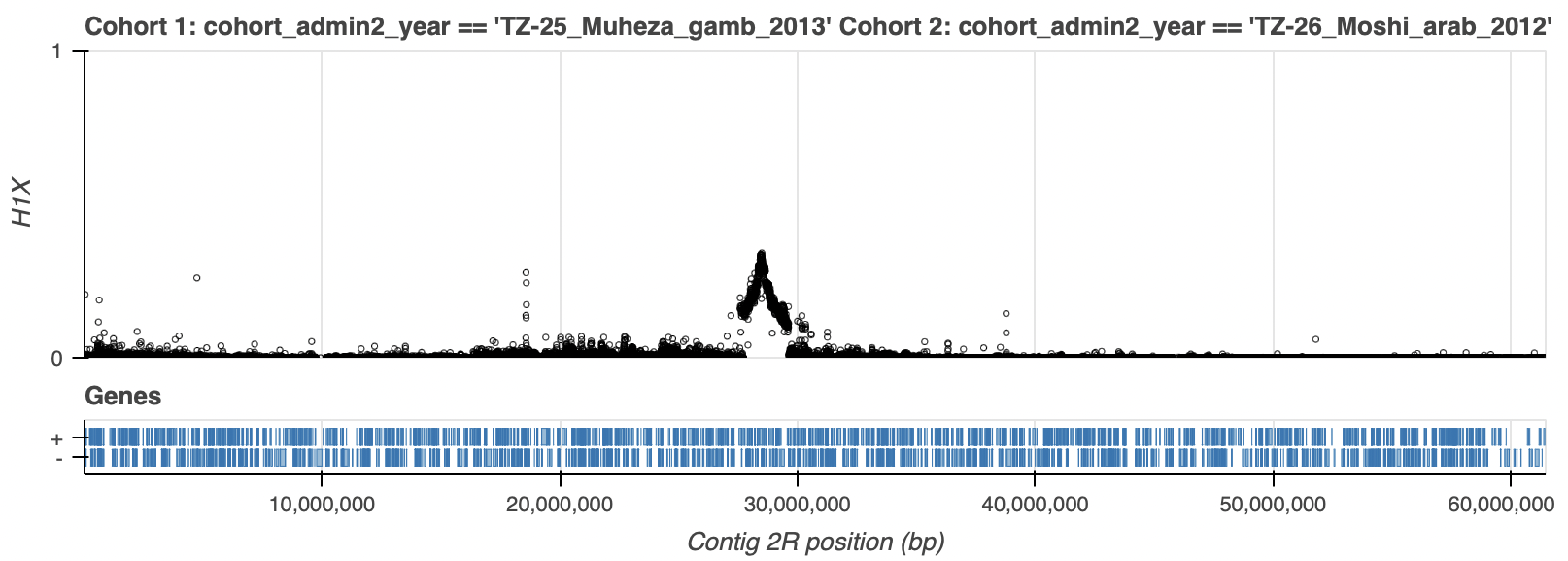

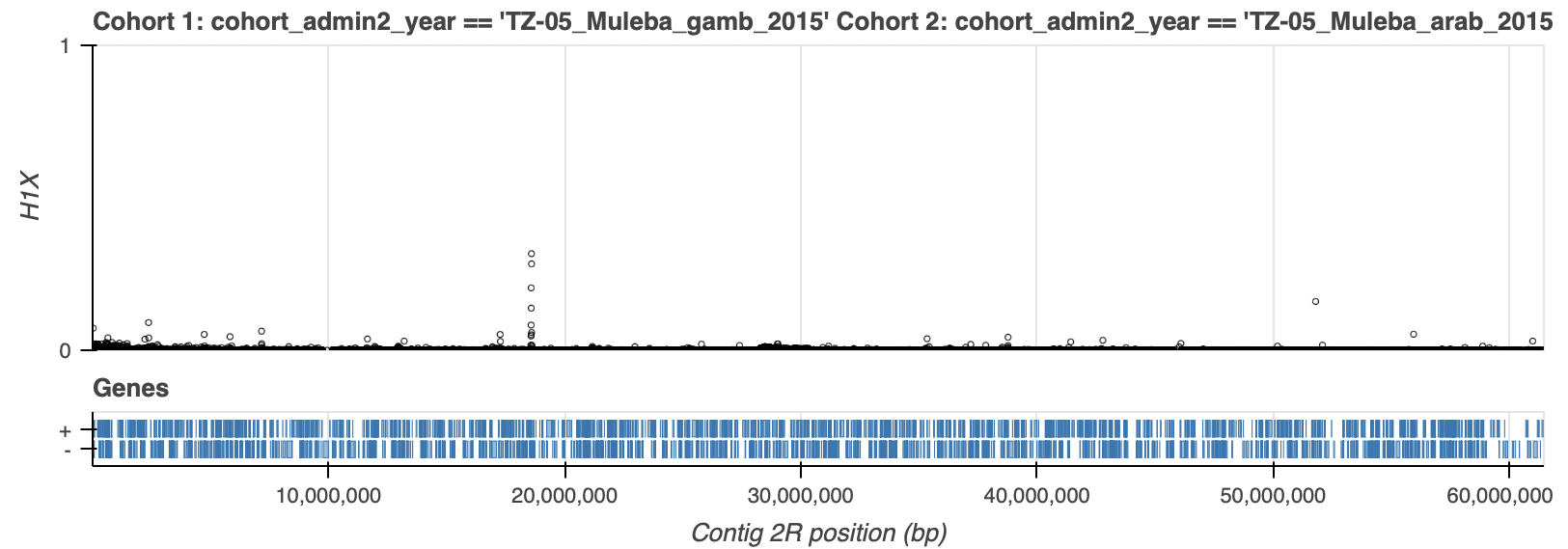

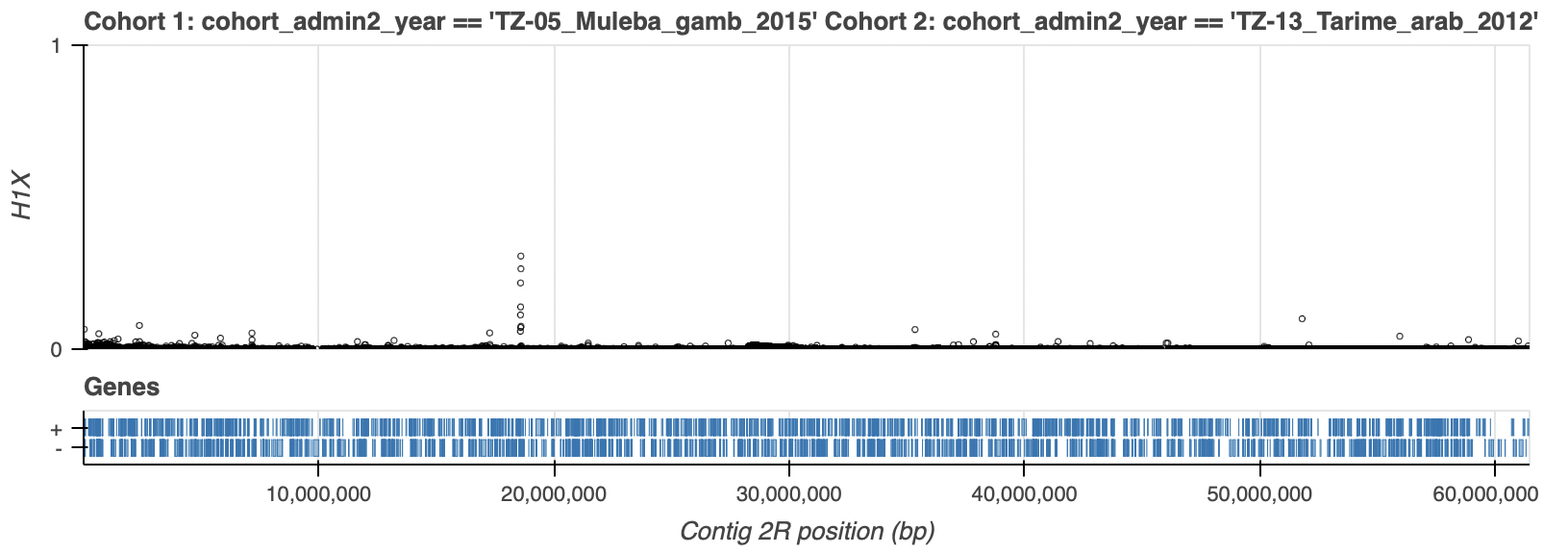

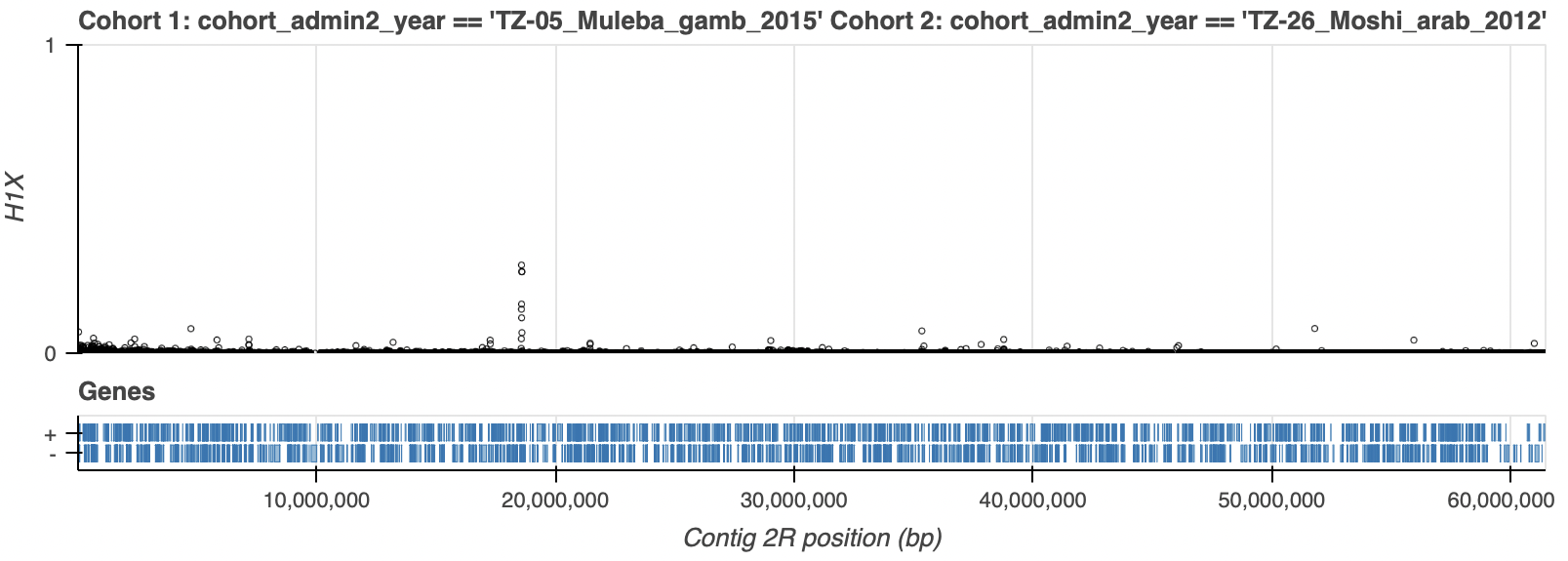


**A**

**B**

**Supplementary 11:** Haplotype clusters showing the spread of resistance haplotypes along the *Cyp6aa/p* loci associated with metabolic resistance, across all taxa of *An. gambiae* complex mosquitoes collected from four selected sites in Northern Tanzania, i.e. *An. arabiensis*, *An. gambiae* s.s. and *Pwani* molecular form.


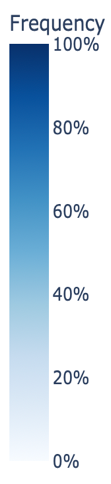

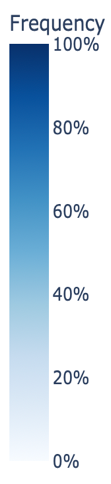

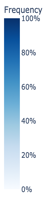

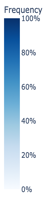

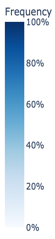


**Supplementary 12:** A heat map showing non-synonymous discordant reads at **A**: *Cyp6aa1* gene, **B**: *Cyp6aa2* gene, **C**: *Cyp6p4* , **D**: *Cyp9k1* and **E**: *Gste2*, the target-site of metabolic resistance in *An. gambiae* complex mosquitoes collected from selected sites in Tanzania.
