# Supplementary material for: Genomic Analysis Reveals a New Cryptic Taxon Within the Anopheles gambiae Complex With a Distinct Insecticide Resistance Profile in the Coast of East Africa
Source: Mol Ecol. 2025 Apr 16;34(10):e17762. doi: 10.1111/mec.17762 (PMC12051790; doi:10.1111/mec.17762)
Supplement: Supplementary file 1 — Figure S1. Figure S2. Figure S3. Figure S4. Figure S5. Figure S6. Figure S7. Figure S8. Figure S9. Figure S10. Figure S11. Figure S12. [file MEC-34-e17762-s001.zip › mec17762-sup-0007-FigureS6.pdf]

Source 1	*Source 2*	*Target*	f_3	*SE*	*Z-score*
Tanzania					
An. gambiae (TZ)	*An. arabiensis* (TZ)	*Pwani* molecular form (TZ)	0.098	0.003	29.576
An. gambiae (TZ)	*An. coluzzii* (CAR)	*Pwani* molecular form (TZ)	0.079	0.003	28.478
An. gambiae (TZ)	*An. melas* (Fontaine)	*Pwani* molecular form (TZ)	0.084	0.003	28.343
An. gambiae (TZ)	*An. quadriannulatus* (Fontaine)	*Pwani* molecular form (TZ)	0.086	0.003	29.776
An. gambiae (TZ)	*An. gambiae* (KE)	*Pwani* molecular form (TZ)	0.083	0.002	39.227
An. gambiae (TZ)	*An. arabiensis* (KE)	*Pwani* molecular form (TZ)	0.098	0.003	29.601
An. gambiae (TZ)	*An. coluzzii* (CAR)	*Pwani* molecular form (TZ)	0.102	0.004	24.908
An. gambiae (TZ)	*An. merus* (Fontaine)	*Pwani* molecular form (TZ)	0.144	0.005	29.482
An. gambiae (TZ)	*An. melas* (Fontaine)	*Pwani* molecular form (TZ)	0.139	0.005	29.091
An. gambiae (TZ)	*An. quadriannulatus* (Fontaine)	*Pwani* molecular form (TZ)	0.143	0.005	30.745
An. gambiae (TZ)	*An. gambiae* (KE)	*Pwani* molecular form (TZ)	0.082	0.003	28.746
An. gambiae (TZ)	*An. arabiensis* (KE)	*Pwani* molecular form (TZ)	0.229	0.003	45.958
Kenya					
An. gambiae (KE)	*An. arabiensis* (TZ)	*Pwani* molecular form (KE)	0.358	0.007	49.611
An. gambiae (KE)	*An. gambiae* (TZ)	*Pwani* molecular form (KE)	0.219	0.006	33.859
An. gambiae (KE)	*An. gambiae* (TZ)	*Pwani* molecular form (KE)	0.216	0.006	36.440
An. gambiae (KE)	*An. coluzzii* (CAR)	*Pwani* molecular form (KE)	0.200	0.006	32.503
An. gambiae (KE)	*An. merus* (Fontaine)	*Pwani* molecular form (KE)	0.200	0.006	31.865
An. gambiae (KE)	*An. melas* (Fontaine)	*Pwani* molecular form (KE)	0.198	0.006	30.888
An. gambiae (KE)	*An. quadriannulatus* (Fontaine)	*Pwani* molecular form (KE)	0.201	0.006	32.210
An. gambiae (KE)	*An. coluzzii* (CAR)	*Pwani* molecular form (KE)	0.225	0.006	36.685
An. gambiae (KE)	*An. merus* (Fontaine)	*Pwani* molecular form (KE)	0.265	0.007	37.757
An. gambiae (KE)	*An. melas* (Fontaine)	*Pwani* molecular form (KE)	0.261	0.007	38.944
An. gambiae (KE)	*An. quadriannulatus* (Fontaine)	*Pwani* molecular form (KE)	0.266	0.008	34.528
