# Supplementary material for: Genomic Analysis Reveals a New Cryptic Taxon Within the Anopheles gambiae Complex With a Distinct Insecticide Resistance Profile in the Coast of East Africa
Source: Mol Ecol. 2025 Apr 16;34(10):e17762. doi: 10.1111/mec.17762 (PMC12051790; doi:10.1111/mec.17762)
Supplement: Supplementary file 1 — Figure S1. Figure S2. Figure S3. Figure S4. Figure S5. Figure S6. Figure S7. Figure S8. Figure S9. Figure S10. Figure S11. Figure S12. [file MEC-34-e17762-s001.zip › mec17762-sup-0008-FigureS7.pdf]

Source 1 (A)	*Source 2 (B)*	*Test (C)*	*Outgroup*	f_4	*SE*	*Z-score*
Pwani molecular form (TZ)	*Pwani* molecular form (KE)	*An. arabiensis* (TZ)	*An. quadriannulatus*	0.022	0.006	3.806
Pwani molecular form (TZ)	*Pwani* molecular form (KE)	*An. arabiensis* (KE)	*An. quadriannulatus*	0.013	0.006	2.203
Pwani molecular form (TZ)	*Pwani* molecular form (KE)	*An. gambiae* (TZ)	*An. quadriannulatus*	0.018	0.004	4.730
Pwani molecular form (TZ)	*Pwani* molecular form (KE)	*An. gambiae* (KE)	*An. quadriannulatus*	0.018	0.004	4.244
Pwani molecular form (TZ)	*Pwani* molecular form (KE)	*An. merus* (Fontaine)	*An. quadriannulatus*	(0.004)	0.007	(0.530)
Pwani molecular form (TZ)	*Pwani* molecular form (KE)	*An. melas* (Fontaine)	*An. quadriannulatus*	0.006	0.007	0.852
Pwani molecular form (TZ)	*Pwani* molecular form (KE)	*An. coluzzii* (CAR)	*An. quadriannulatus*	(0.030)	0.004	(7.039)
