# Supplementary figures and images for: Genomic Analysis Reveals a New Cryptic Taxon Within the Anopheles gambiae Complex With a Distinct Insecticide Resistance Profile in the Coast of East Africa
Source: Mol Ecol. 2025 Apr 16;34(10):e17762. doi: 10.1111/mec.17762 (PMC12051790; doi:10.1111/mec.17762)

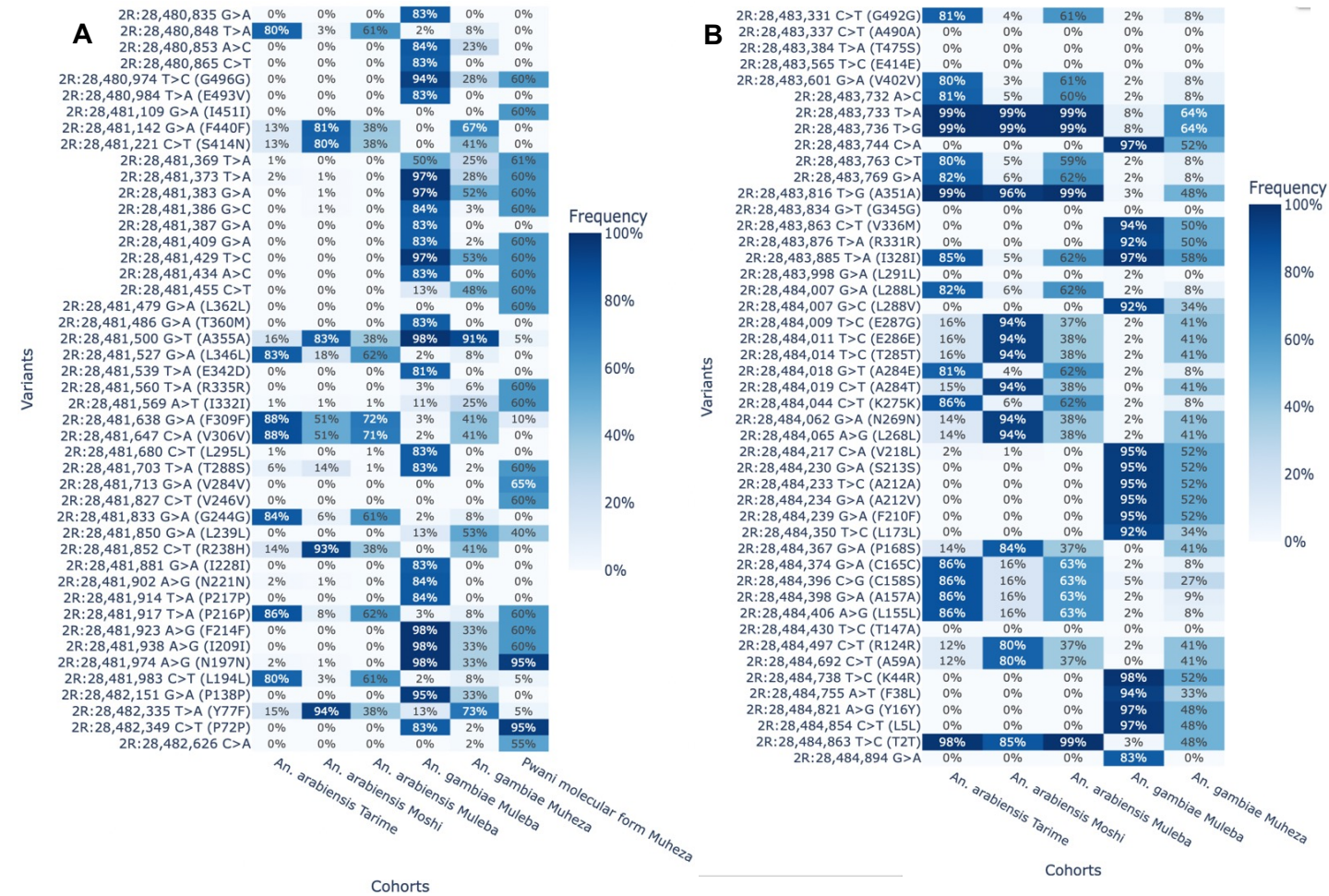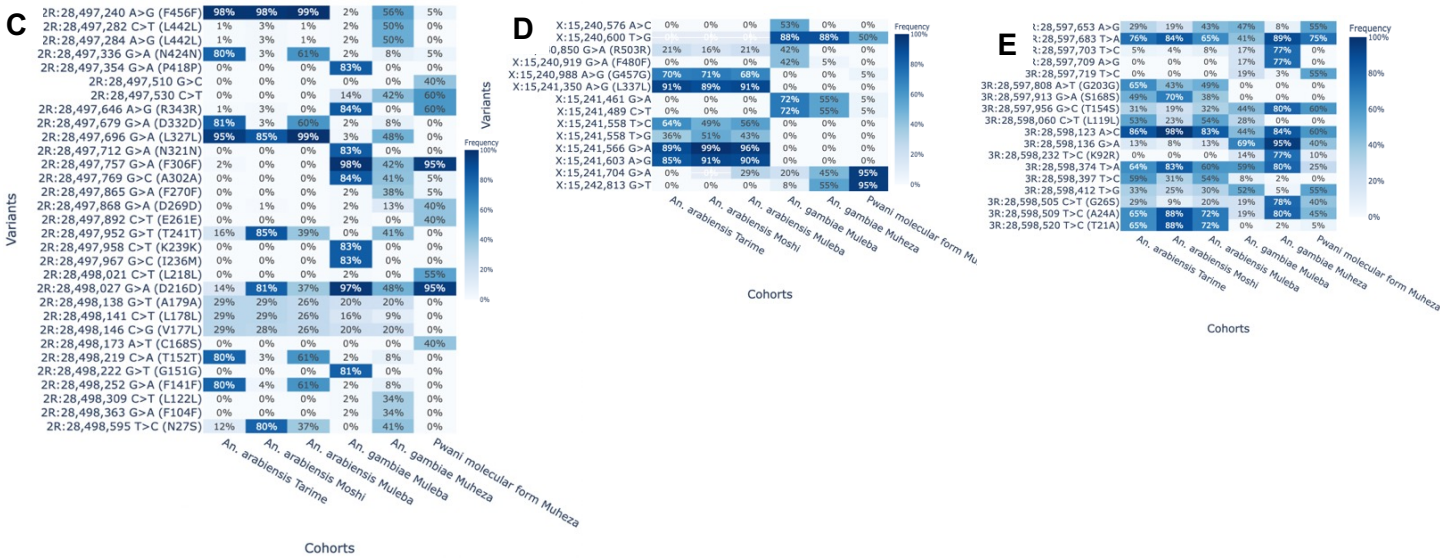

Supplement: Supplementary file 1 — Figure S1. Figure S2. Figure S3. Figure S4. Figure S5. Figure S6. Figure S7. Figure S8. Figure S9. Figure S10. Figure S11. Figure S12. [file MEC-34-e17762-s001.zip › mec17762-sup-0013-FigureS12.pdf]

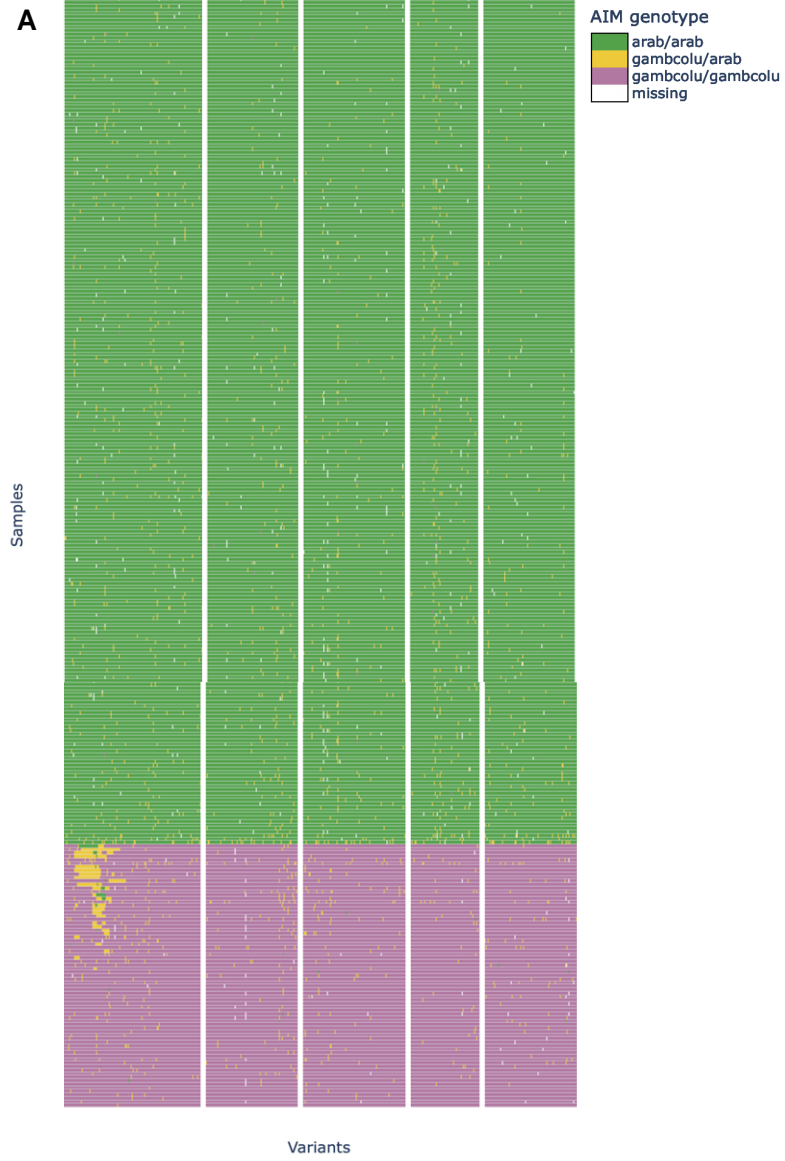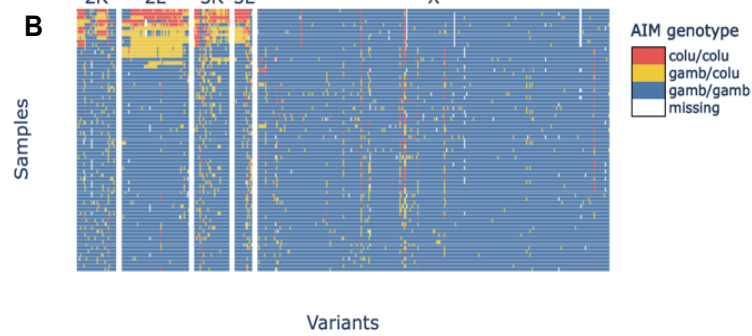

Supplement: Supplementary file 1 — Figure S1. Figure S2. Figure S3. Figure S4. Figure S5. Figure S6. Figure S7. Figure S8. Figure S9. Figure S10. Figure S11. Figure S12. [file MEC-34-e17762-s001.zip › mec17762-sup-0002-FigureS1.pdf]

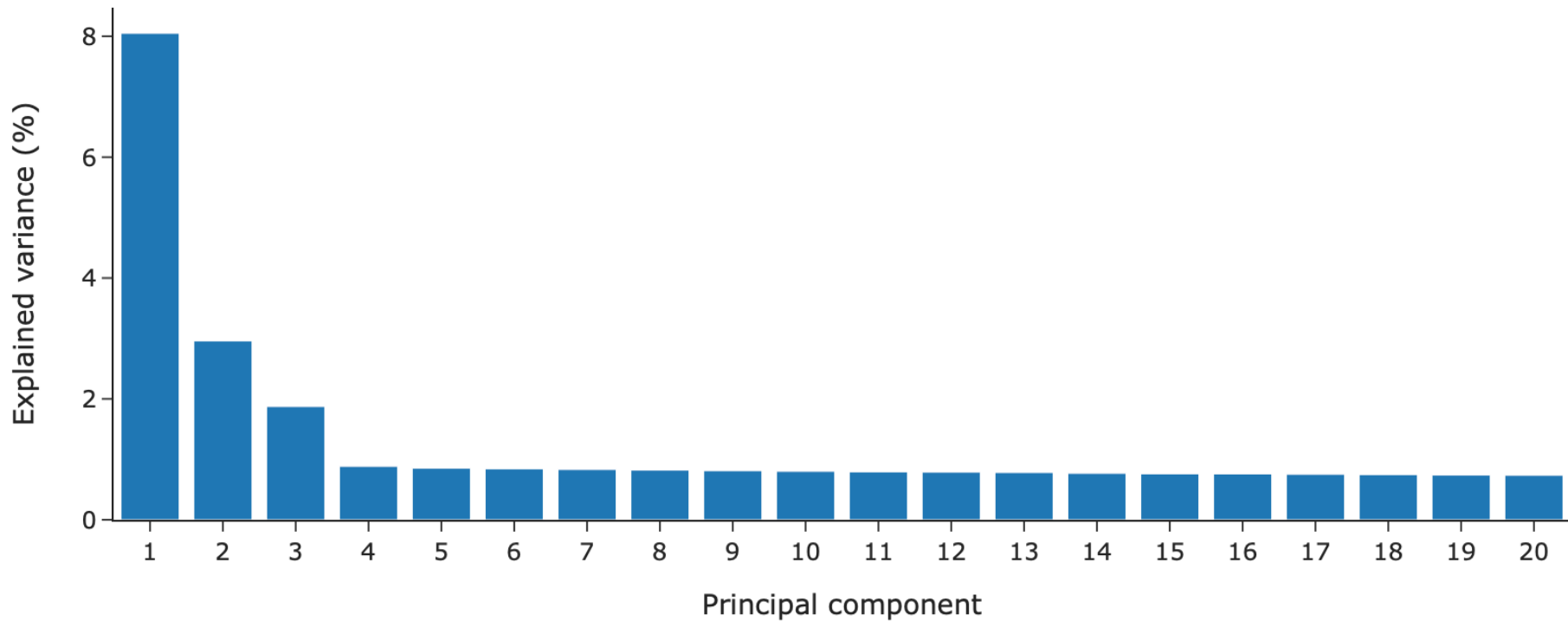

Supplement: Supplementary file 1 — Figure S1. Figure S2. Figure S3. Figure S4. Figure S5. Figure S6. Figure S7. Figure S8. Figure S9. Figure S10. Figure S11. Figure S12. [file MEC-34-e17762-s001.zip › mec17762-sup-0003-FigureS2.pdf]

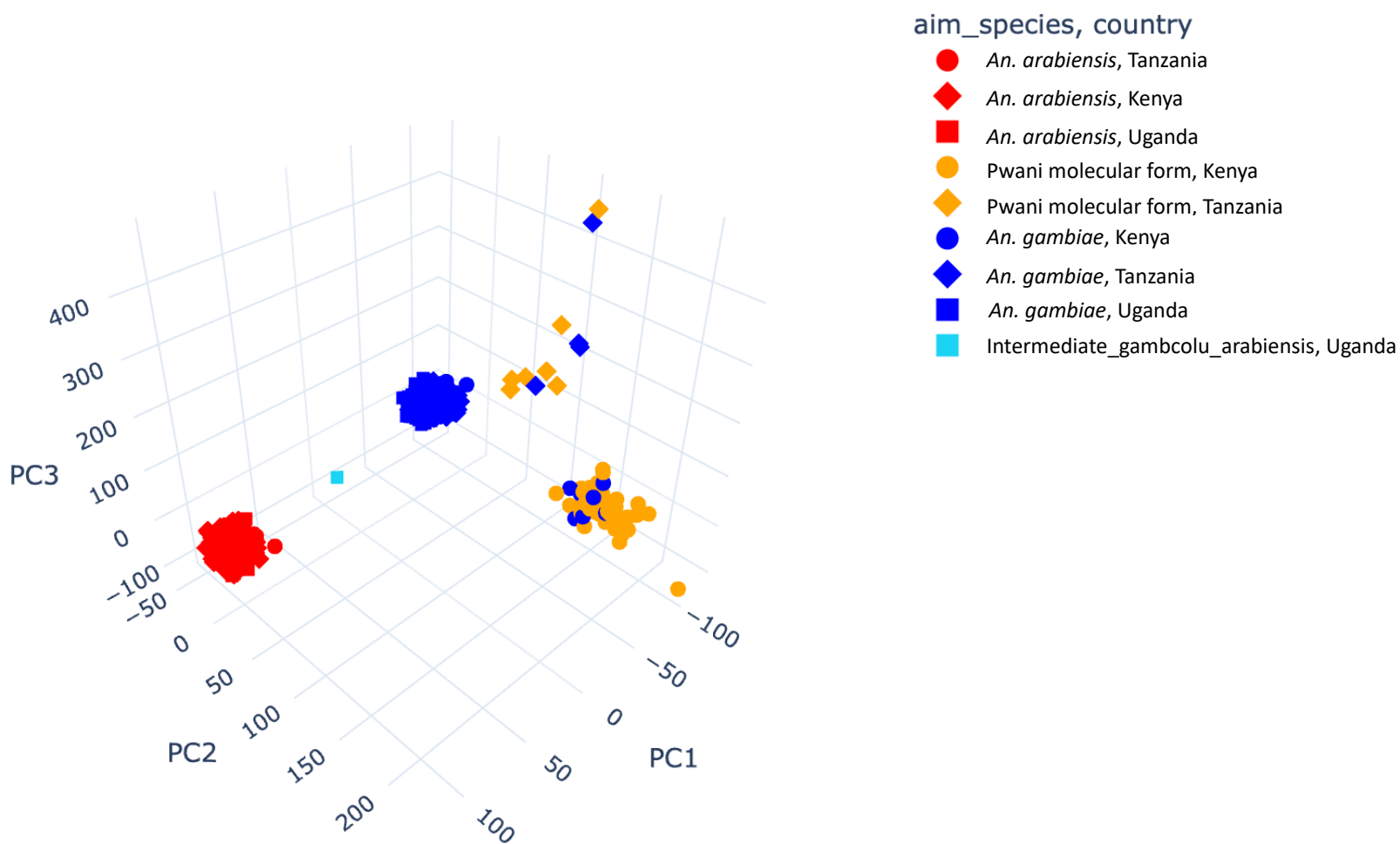

Supplement: Supplementary file 1 — Figure S1. Figure S2. Figure S3. Figure S4. Figure S5. Figure S6. Figure S7. Figure S8. Figure S9. Figure S10. Figure S11. Figure S12. [file MEC-34-e17762-s001.zip › mec17762-sup-0004-FigureS3.pdf]

Nucleotide diversity

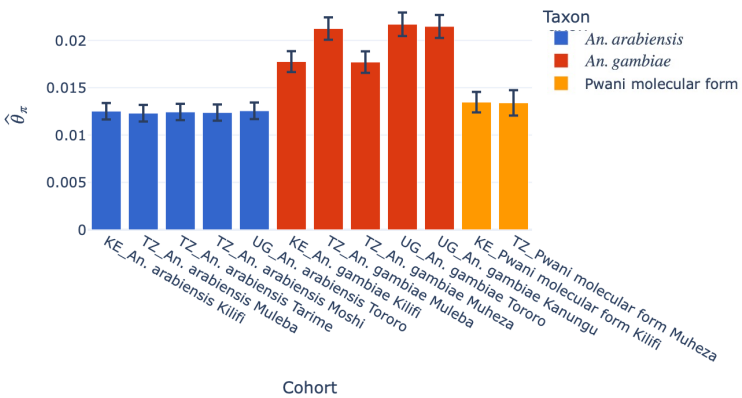

Tajima's D

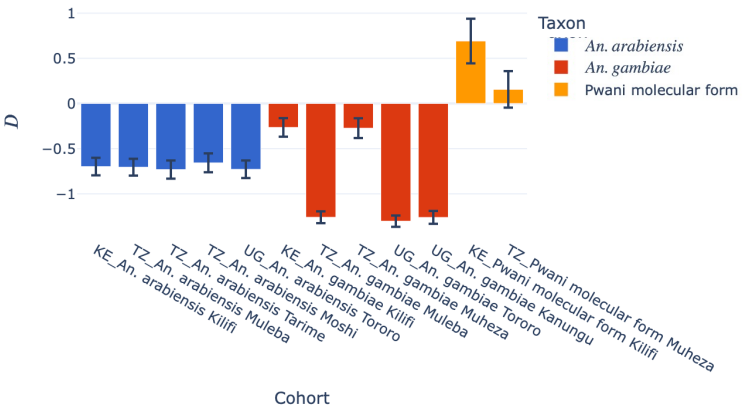

A Watterson estimator

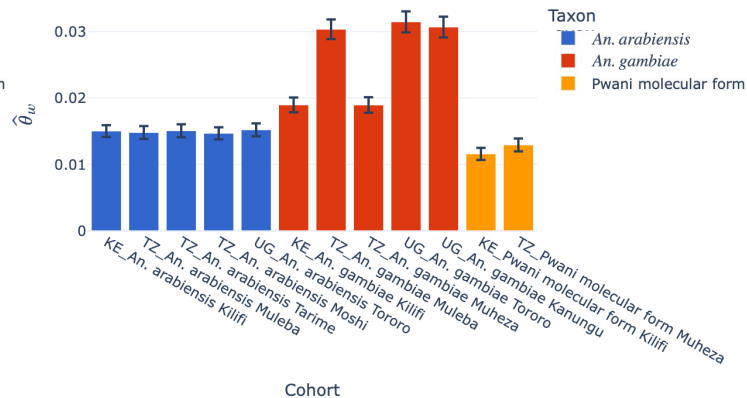

C

Diversity estimators

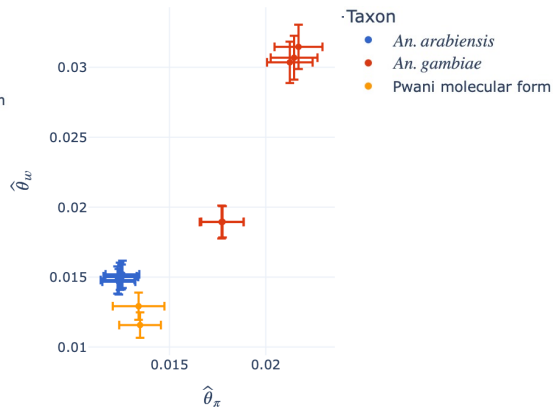

B

D

Supplement: Supplementary file 1 — Figure S1. Figure S2. Figure S3. Figure S4. Figure S5. Figure S6. Figure S7. Figure S8. Figure S9. Figure S10. Figure S11. Figure S12. [file MEC-34-e17762-s001.zip › mec17762-sup-0005-FigureS4.pdf]

$F_{st}$  values

0.35  
0.30  
0.25  
0.20  
0.15  
0.10  
0.05  
0.00

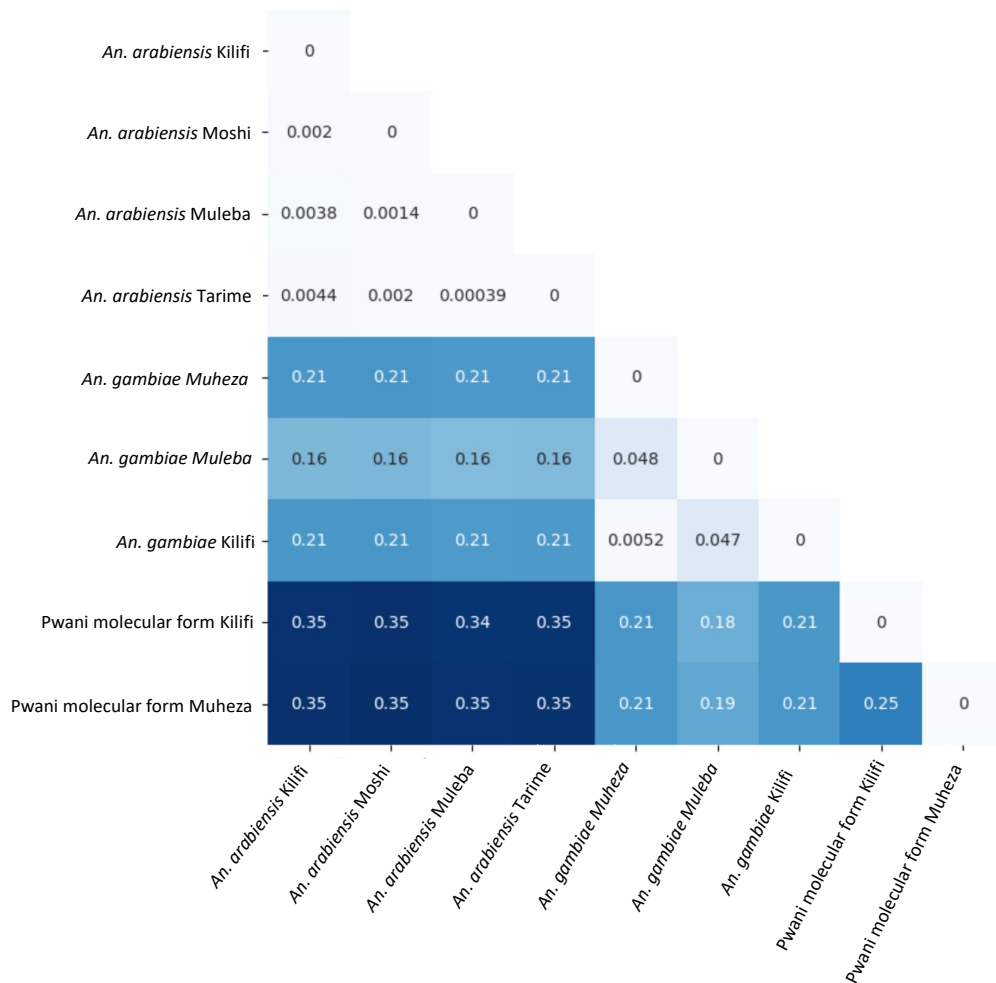

Supplement: Supplementary file 1 — Figure S1. Figure S2. Figure S3. Figure S4. Figure S5. Figure S6. Figure S7. Figure S8. Figure S9. Figure S10. Figure S11. Figure S12. [file MEC-34-e17762-s001.zip › mec17762-sup-0006-FigureS5.pdf]

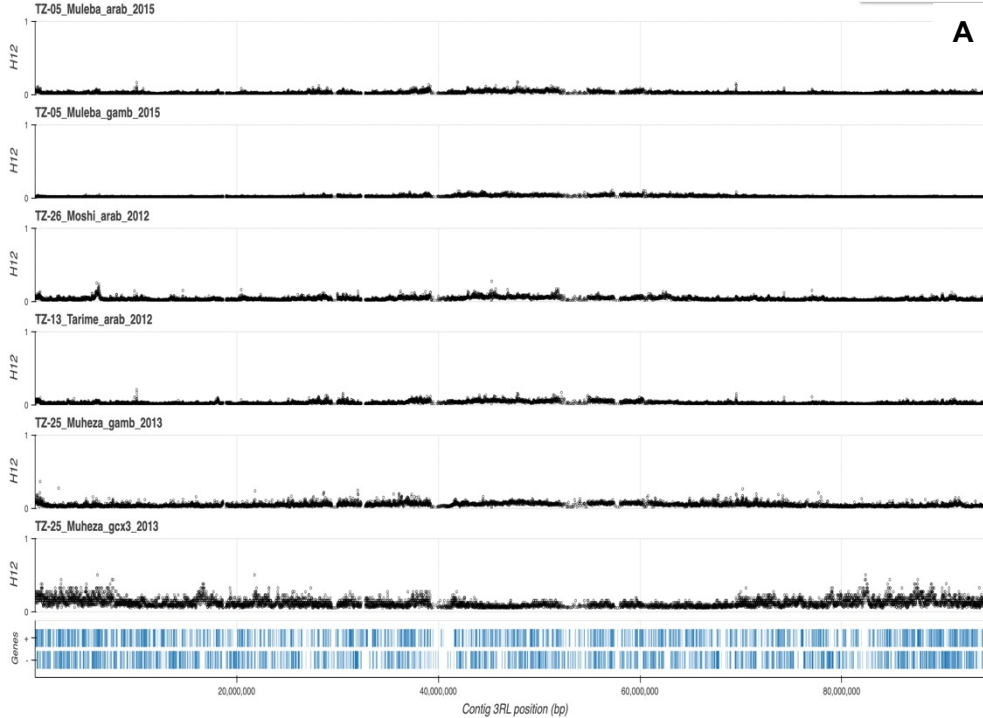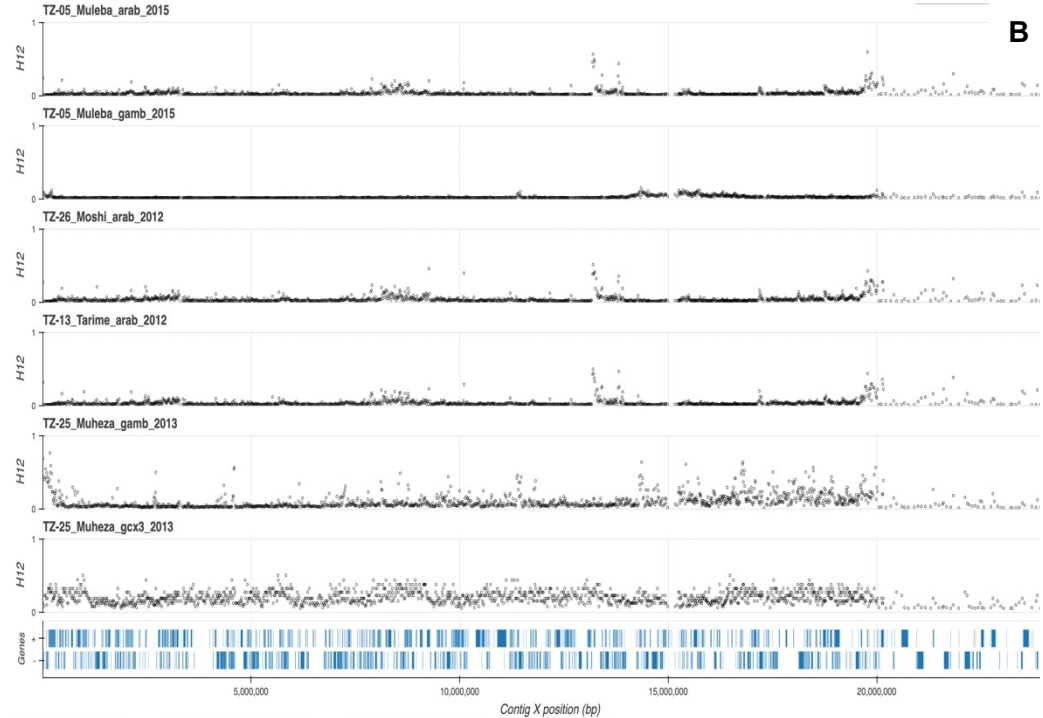

Supplement: Supplementary file 1 — Figure S1. Figure S2. Figure S3. Figure S4. Figure S5. Figure S6. Figure S7. Figure S8. Figure S9. Figure S10. Figure S11. Figure S12. [file MEC-34-e17762-s001.zip › mec17762-sup-0009-FigureS8.pdf]

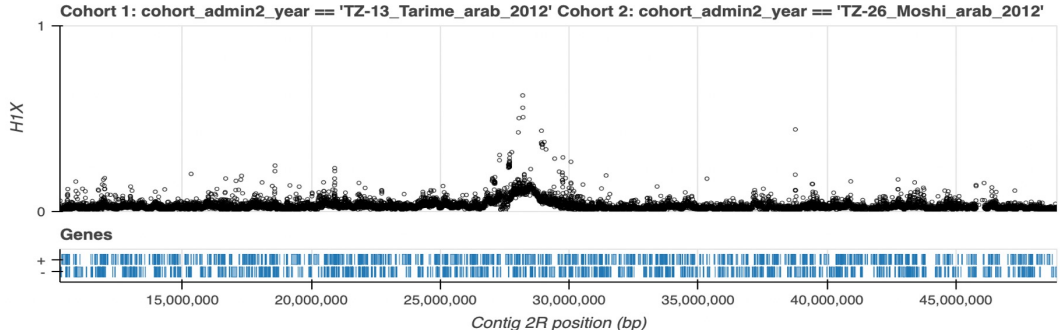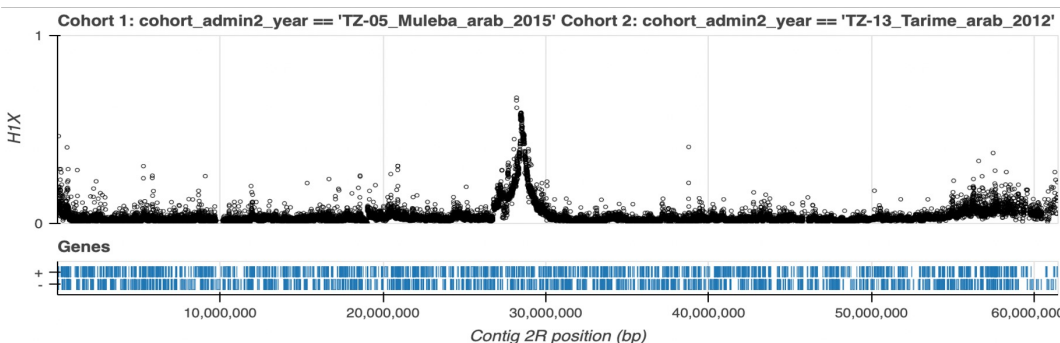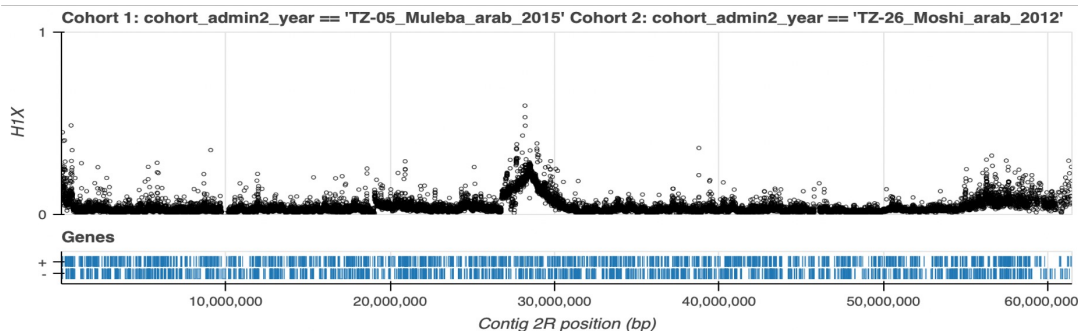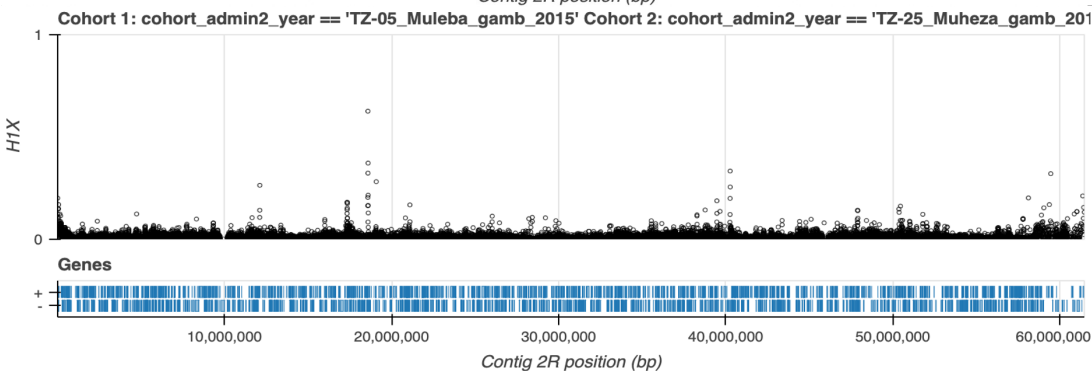

*An. arabiensis*  
cohorts

**A**

*An. gambiae*  
cohorts

**B**

Supplement: Supplementary file 1 — Figure S1. Figure S2. Figure S3. Figure S4. Figure S5. Figure S6. Figure S7. Figure S8. Figure S9. Figure S10. Figure S11. Figure S12. [file MEC-34-e17762-s001.zip › mec17762-sup-0010-FigureS9.pdf]

**A**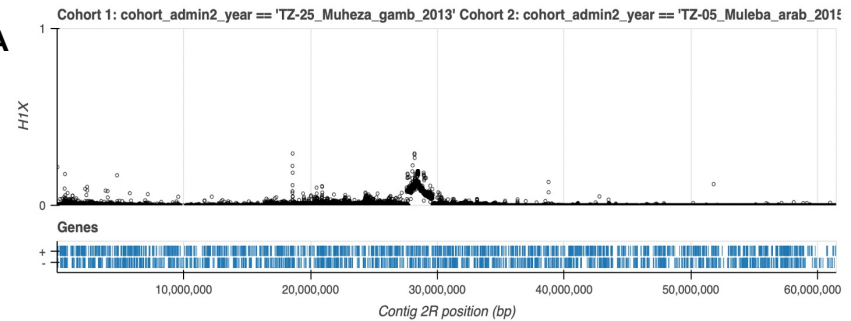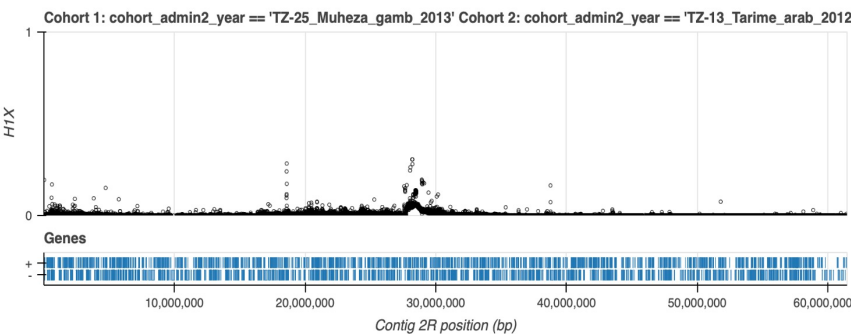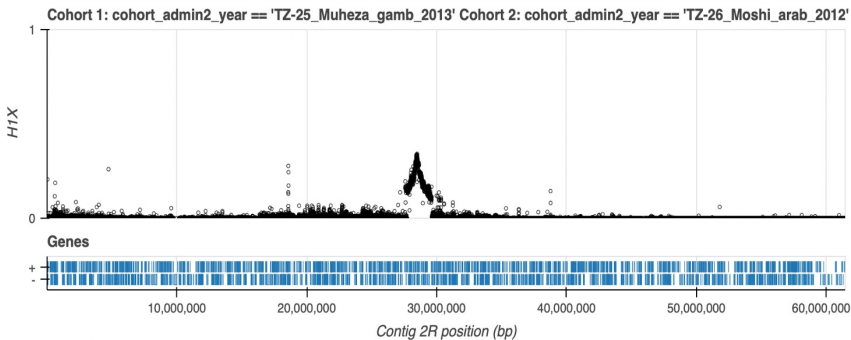**B**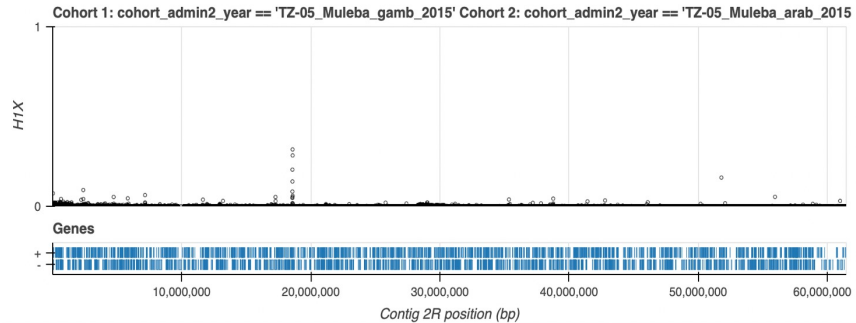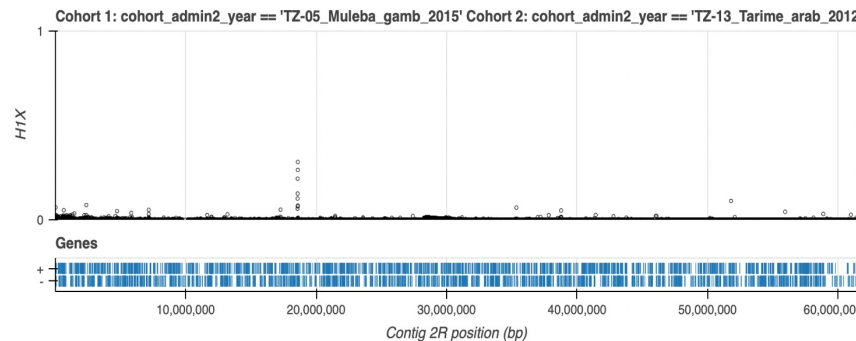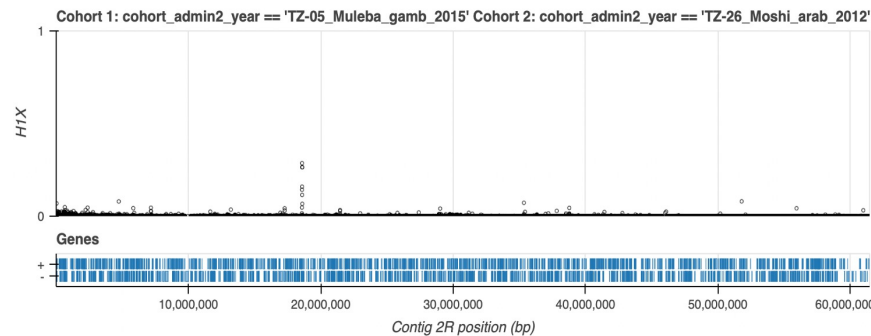

Supplement: Supplementary file 1 — Figure S1. Figure S2. Figure S3. Figure S4. Figure S5. Figure S6. Figure S7. Figure S8. Figure S9. Figure S10. Figure S11. Figure S12. [file MEC-34-e17762-s001.zip › mec17762-sup-0011-FigureS10.pdf]
